# Nano-priming as emerging seed priming technology for sustainable agriculture—recent developments and future perspectives

**DOI:** 10.1186/s12951-022-01423-8

**Published:** 2022-06-03

**Authors:** Shivraj Hariram Nile, Muthu Thiruvengadam, Yao Wang, Ramkumar Samynathan, Mohammad Ali Shariati, Maksim Rebezov, Arti Nile, Meihong Sun, Baskar Venkidasamy, Jianbo Xiao, Guoyin Kai

**Affiliations:** 1 Laboratory for Core Technology of TCM Quality Improvement and Transformation, The Third Affiliated Hospital, School of Pharmaceutical Science, Zhejiang Chinese Medical University, Hangzhou, Zhejiang 310053 People’s Republic of China; 2 Department of Crop Science, College of Sanghuh Life Science, Konkuk University, Seoul, 05029 Republic of Korea; 3 Institute of Plant Biotechnology, School of Life Sciences, Shanghai Normal University, Shanghai, 200234 People’s Republic of China; 4 R&D Division, Alchem Diagnostics, No. 1/1, Gokhale Street, Ram Nagar, Coimbatore, 641009 Tamil Nadu India; 5 Scientific Department, K.G. Razumovsky Moscow State University of Technologies and Management (The First Cossack University), 73, Zemlyanoy Val St., Moscow, 109004 Russian Federation; 6 Department of Scientific Research, V. M. Gorbatov Federal Research Center for Food Systems, 26 Talalikhina St., Moscow, 109316 Russian Federation; 7 Department of Biotechnology, Sri Shakthi Institute of Engineering and Technology, Coimbatore, 641062 Tamil Nadu India; 8 Department of Analytical Chemistry and Food Science, Faculty of Food Science and Technology, University of Vigo, Vigo, Spain; 9 Laboratory of Medicinal Plant Biotechnology, College of Pharmacy, Zhejiang Chinese Medical University, Hangzhou, Zhejiang 310053 People’s Republic of China

**Keywords:** Nanoparticles, Reactive oxygen species, Seed germination, Plant metabolism, Sustainable agriculture

## Abstract

Nano-priming is an innovative seed priming technology that helps to improve seed germination, seed growth, and yield by providing resistance to various stresses in plants. Nano-priming is a considerably more effective method compared to all other seed priming methods. The salient features of nanoparticles (NPs) in seed priming are to develop electron exchange and enhanced surface reaction capabilities associated with various components of plant cells and tissues. Nano-priming induces the formation of nanopores in shoot and helps in the uptake of water absorption, activates reactive oxygen species (ROS)/antioxidant mechanisms in seeds, and forms hydroxyl radicals to loosen the walls of the cells and acts as an inducer for rapid hydrolysis of starch. It also induces the expression of aquaporin genes that are involved in the intake of water and also mediates H_2_O_2,_ or ROS, dispersed over biological membranes. Nano-priming induces starch degradation via the stimulation of amylase, which results in the stimulation of seed germination. Nano-priming induces a mild ROS that acts as a primary signaling cue for various signaling cascade events that participate in secondary metabolite production and stress tolerance. This review provides details on the possible mechanisms by which nano-priming induces breaking seed dormancy, promotion of seed germination, and their impact on primary and secondary metabolite production. In addition, the use of nano-based fertilizer and pesticides as effective materials in nano-priming and plant growth development were also discussed, considering their recent status and future perspectives.

**Graphical Abstract:**

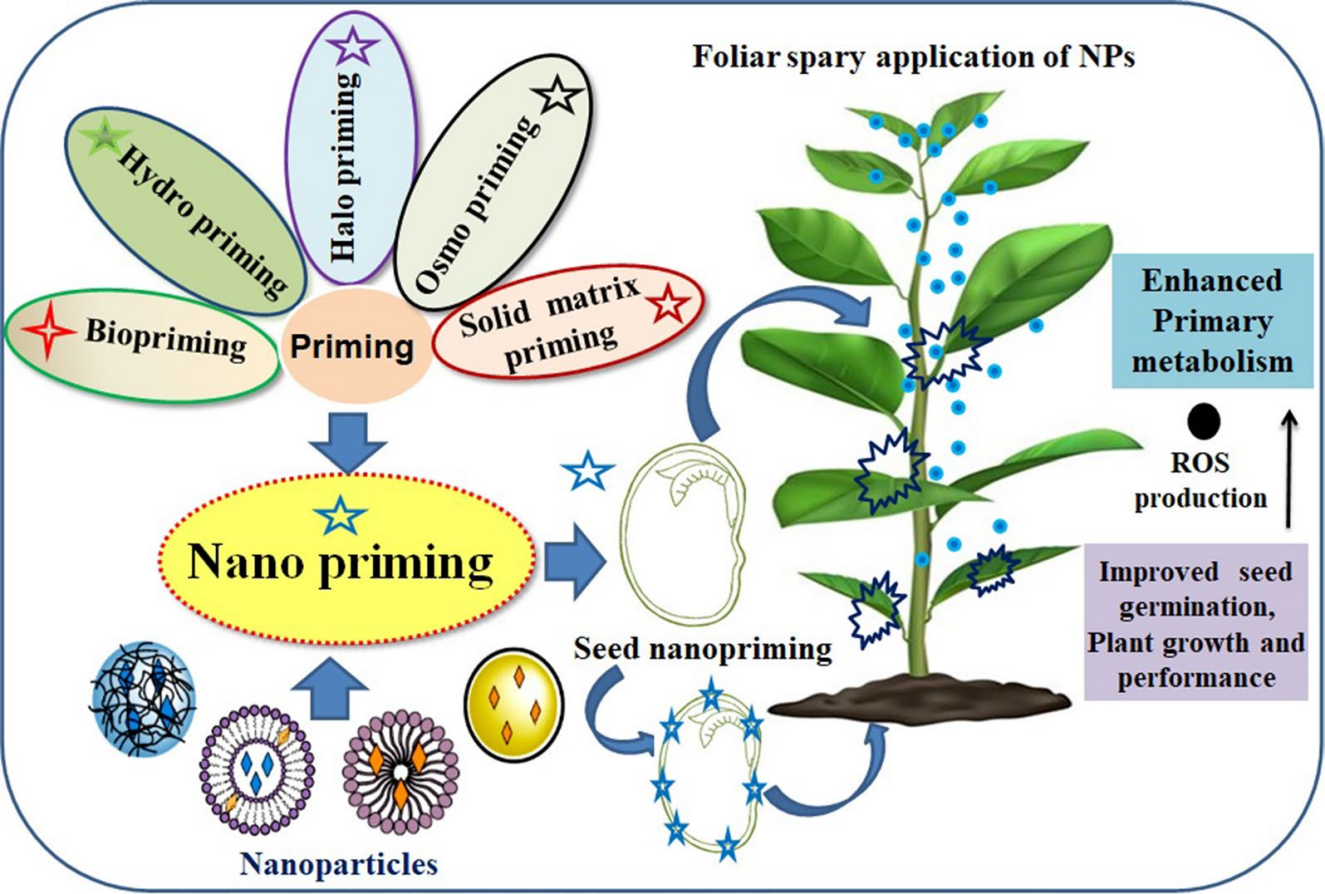

## Background

**Fig. 1:**
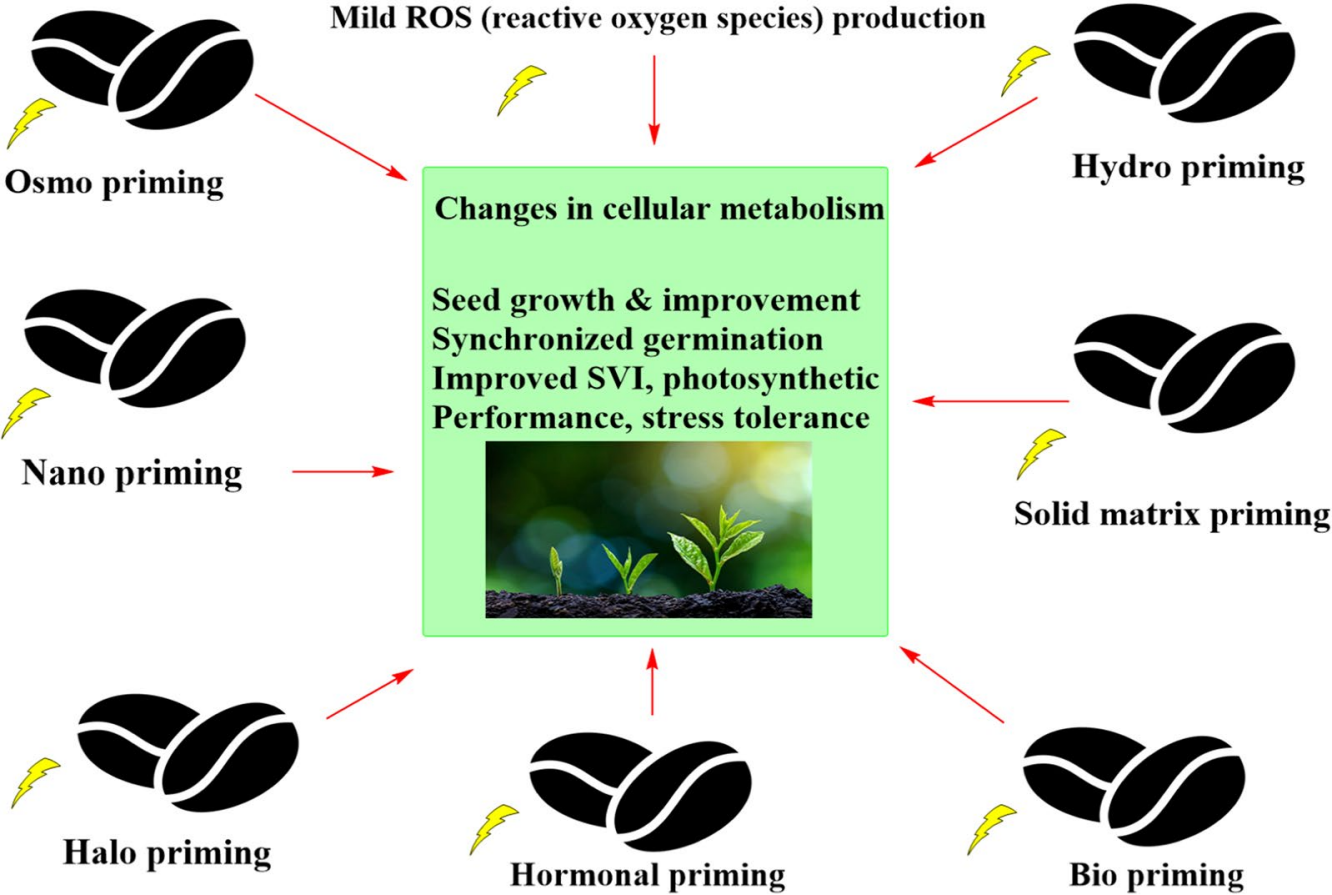
Impact of different types of seed priming in the growth and physiological changes in plants

Seed priming is a pre-sowing treatment that causes a physiological change in the seed that permits it to germinate more rapidly [1]. It also enhances crop activity by stimulating the resistance of plants against abiotic and biotic stresses [2]. Priming is the process of pre-treating seeds before planting those plants using traditional methods such as pre-soaking and coating. Alternative seed hydration methods (with or without aeration) are used in the pre-germination phase, and seeds are then re-dried to the usual moisture content of the regular handling process such as sowing, packaging, and preservation. Seed dormancy can be reduced by treating the seeds in salt solutions (halo-priming), water (hydro-conditioning), osmotic agents (osmo-priming), plant hormone solutions (hormonal priming), valuable microbe solutions (bio-priming), under a magnetic field (magneto-priming), a solution containing a solid carrier (matri-conditioning), and solutions containing nanoparticles (NPs) (nano-priming) [3, 4]. Hydro-priming, osmo-priming, hormonal priming, nutri-priming, on-farm priming, and bio-priming are examples of current priming processes that have shown potential benefits for crops, including improved germination rates, germination energy, growth and development, increased abiotic and biotic stress tolerance, and increased crop yield and micronutrient concentrations in cereals [5]. Furthermore, different priming agents have a variety of distinct traits and possibilities, and they should be optimised for each crop species. Priming using nanoparticles (nano-priming) has been proven to be more promising than traditional priming approaches for achieving feasible agricultural yields.[6]. Nano-priming uses nanoparticles (NPs) with a size of less than 100 nm, and "priming" relates to the development of stress tolerance under moderate and recurring stress [7]. Different types of Nano-priming were shown in Fig. 1. It has been reported that seed germination and seedling vigor are potentially induced in various crops upon nano-priming [7, 8, 9]. Moreover, this may be one of the best methods to sort out the dormancy problems and increase the germination of seeds in forest species (upland boreal), which indicates that nano-priming can be useful for forest reclamation purposes [10]. However, many studies have demonstrated that high quantities of NPs can have toxicological effects on crops including lettuce, tomato, wheat, and cucumber [11]. The induction of plant secondary metabolism to offset the adverse environmental stresses results in the synthesis of several types of plant secondary metabolites like phenylpropanoids, alkaloids, sulphur-compounds (including glucosinolates) and terpenoids. In addition to their role in a/biotic stress tolerance, they have been utilized as bioactive compounds (antioxidants, anticancer, antimicrobials, etc.) and provide protection against various diseases [12–14].

The regulated delivery of nano-encapsulated materials (pesticides & fertilizers) on-site represents a comprehensive alternative for improving crops, the surrounding habitat and animal health. There is an ongoing effort to generate nano-agrochemicals to control the release of certain nutrients, thereby preserving the fertility and health of the soil [15]. Although nano-priming might be an effective approach to induce seed germination and attribute tolerance to mild and intermittent stresses, it has not been studied in detail. The studies on nano-priming in plants have been considered as an emerging field and the available scanty reports induce us to provide a review of the potential of nano-priming in crop plants. This review briefly describes the nano-priming approaches, their mechanisms, and their potential benefits for crops.

### General mode of entry and mechanisms of actions of NPs

The plasma membrane has a phospholipid bilayer with hydrophilic head groups, and hydrophobic tails acting as a barricade for the transfer of molecules. The entry of NPs into the cells is proposed by three mechanisms [16]. According to the first mechanism, NPs are small molecules that can easily cross the plasma membrane by a direct diffusion process. The passage is concerned with numerous features, namely the size, hydrophobicity, constitution, charge, and shape of the particles [17]. In the second mechanism, NPs are actively transported into the cell by engulfing its cell membrane, a process called endocytosis [18]. The third mechanism is by means of transmembrane proteins or through channels that regulate the movement of NPs into cells [19]. Nevertheless, the NP entrance is limited by distinct factors, namely the high degree of specificity, the least open possibility, and small pores [20]. The mode of transport of NPs from the plant cell to the tissues is through foliar/shoots or roots. This transfer is mediated by either an apoplastic or symplastic route of transport [21]. It is quite interesting to know the way of transport of NPs into various parts of the plant. NPs are mainly mobilized from the root system through the xylem to the shoot and no downward movement occurs.

Contrarily, foliar spray of NPs translocated through phloem and accumulates in plant organs [22]. Apoplastic transport occurs on the outer plasma membrane via the extracellular matrix, xylem vessels, and cell walls of neighboring cells. It allows NPs to move radially to reach the vascular tissue and root central cylinder and then ascends to the plant's above-ground parts [23]. Whereas symplastic transport involves the movement of water and other substances between the adjacent cells through plasmodesmata and sieve plates [24]. Stanley et al. suggested that seed priming is the process of rapidly imbibing a small amount of water into seeds to initiate the pre-requisite metabolic activities for pre-germination without radical protrusion [24]. By restricting the movement of water to complete the radical protrusion, the priming process can extend the lag phase and also inhibit the start of the log phase. Ma et al. found that the diffusion of NPs to cotyledons is aided by their entry into seeds through the intercellular spaces of parenchymatous tissue [25]. According to Guha et al. [26], the acquisition of NPs on the seed coat causes the accumulation of reactive oxygen species (ROS), which activates numerous chains of downstream activities [26]. According to Oracz and Karpinski, the spatial and temporal localization of ROS is important for cell-to-cell communication and the breakage of hydrolytic bonds between polysaccharides in the seed endosperm cell wall [27]. Xu et al. investigated *Suaeda salsa* seeds and observed that seed coat phenolic is endogenously regulated by the hormonal balance of abscisic acid (ABA) and gibberellic acid (GA), facilitating nutrient passage across seed compartments [28]. Most of the NPs were found to be taken up and accumulated in plants through roots, leaves and root hairs.

**Fig. 2:**
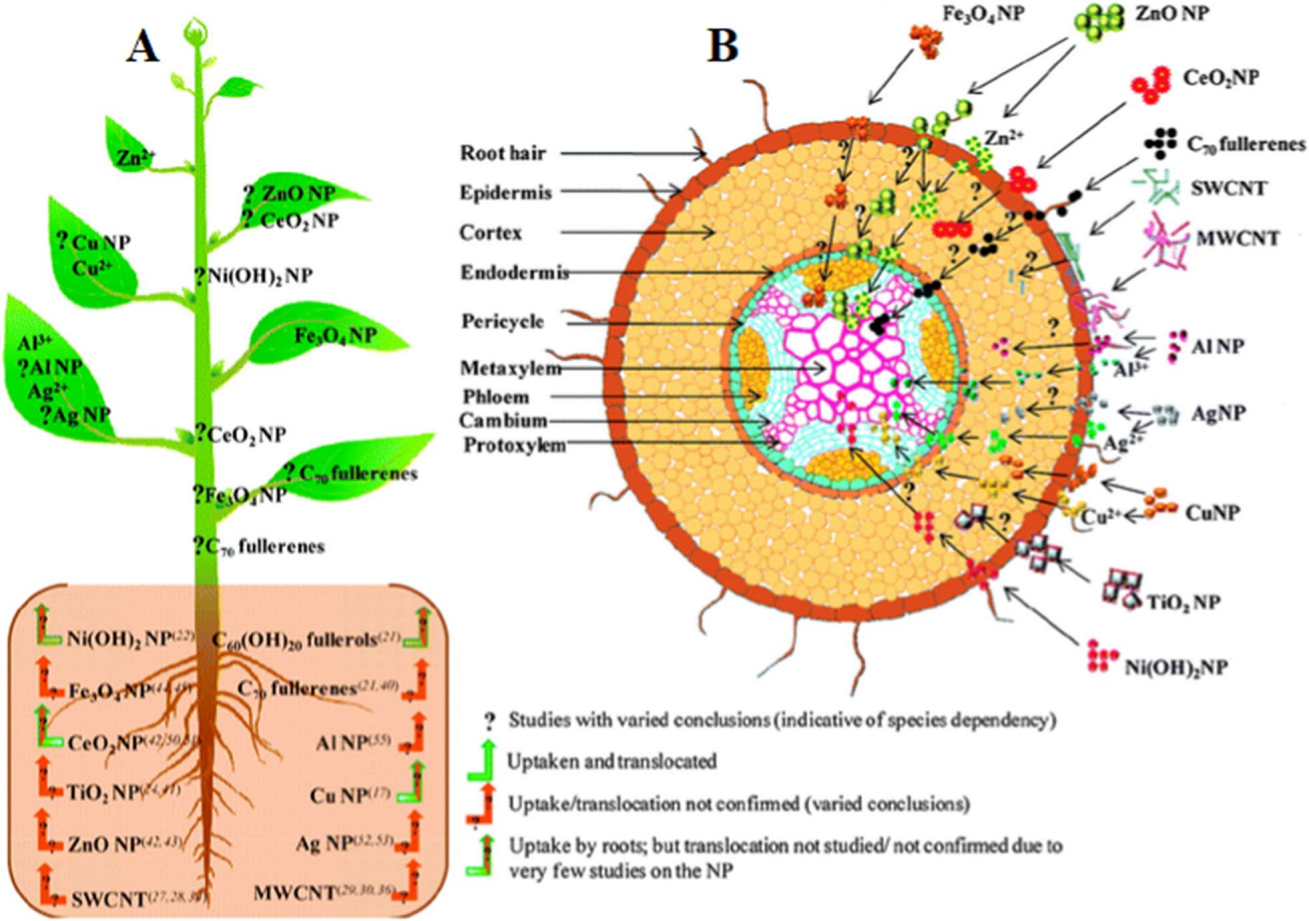
The plants showing selective uptake, regulation, biotransformation, distribution and translocation of different kinds of nanoparticles, **A** Plant showing the selective uptake and translocation of nanoparticles. **B** transverse cross-section of the root absorption zone showing the differential nanoparticle interaction on exposure. This figure was modified and adapted from reference [20] with permission. Copyright 2011 American Chemical Society

The plasmodesmata [29] and the carrier proteins called aquaporins [30] facilitate NPs entry into the cells of the plants. Zhai et al. demonstrated that gold nanoparticles (AuNPs) can be delivered through plasmodesmata [31]. Ion channels [32], cuticle membrane and stomata [33], vasculature [34] are also possible ways of transporting of NPs into plant cells. NPs can also enter plants through a variety of entry points, including cell wall junctions, pit membranes, hydathodes, casparian strips, and extracellular spaces [35]. Throughout the scenario of a second system of entering the NPs into the plant, the possible pathways of NPs entering the roots through rhizoderms, lateral roots, tips and hairs of roots [36, 37]. High ionic strength can lead to fast aggregation of NPs, while organic macromolecules (such as humic acid, fulvic acid, citric acid, and extracellular polymeric substances) can enhance NP stability and reduce sedimentation and/or deposition [38]. Other low-molecular weight organic acids in root exudates, such as citrate and malate, also complex Fe and Cu, to solubilize minerals. Increased solubility through complexation can drive uptake into the plant or root-associated bacteria. For example, citrate complexes Cu and enhances Cu uptake by the root colonizer *Pseudomonas putida* [39]. The concentrations of organic ligands in the root exudates, which play a crucial role in metal bioavailability in the rhizosphere, increase with higher doses of Cu ions [40]. The plant parts show selective uptake, regulation, biotransformation, distribution and translocation of different kinds of nanoparticles as schematically presented in Fig. 2.

### Adhesions and crosstalk between NPs induced ROS and phytohormones in breaking seed dormancy

Seed dormancy is referred to as the seeds are incapable to complete germination even under favorable environmental situations. It’s known as an adaptive trait preventing premature germination out of season. The physical dormancy of seed is an adaptive feature that is generally present in higher plants. This kind of dormancy is imposed by a water-impermeable layer that impedes water and oxygen from the surrounding environment and keeps embryos in a viable condition for a long time [41]. There are three major types of seed dormancy: (a) harder seed coats, in which water and gas (physical dormancy) cannot permeate and thus dormancy can be reduced mainly through mechanical or chemical processes, creating cracks in the plants [42](b) Embryos or internal dormancy are caused through endogenous plant hormones namely cytokinin, ethylene, indole-3-acetic acid, abscisic acid (ABA) and the cells stop growing (c) primary seed dormancy is stimulated by germination suppressors, including cis-ABA; -d- lucopyranosyl ester; benzoic acid; salicylic acid; chlorogenic acid; and coumarin, etc., which prevent germination and harvesting"[43].

Nano-priming was found to be effective where seed vigour index (SVI) measurement was performed and was proven to improve seed cell membrane integrity, thereby increasing the phosphorylation efficiency [5]. Seed priming with micronutrient NPs revealed as a new promising mechanism for improving the rate of seed germination, seedling vigor, and development [44]. The increased perforation of water and nutrients via the seed coat in nano-priming seeds enhanced the growth rate and germination of seedlings [45]. The binding capacity of the nano-primed seeds was superior to hydro, vitamins and polyethylene glycol (PEG) primed seeds [46, 47]. In general, the enhanced water uptake was usually noticeable in nano-priming treatments [48]. The uptake of water by the seeds is impacted by the equilibrium amidst ABA and GA that controls on/off dormancy, in turn, changes in water capability thresholds for radicle development [49]. Moreover, ABA and GA hormonal balance control the phenolics in the seed coat and aid in nutrient transport beyond the compartments in *S. salsa* seeds [28]. While it was also shown that IAA was synthesized in the endosperm, passages to the seed coat in crosstalk with GA with the help of the *AGL62* transcription factor (TF) [50]. The role of IAA in the internalization and movement of NPs in nano-primed seeds and its crosstalk with other hormones (ABA/GA) in inducing the attachment potential of NPs should be clarified by analyzing the auxin deficient mutants (*msg1*; defective in hypocotyl growth) in nano-priming and non-priming treatments [51]. Yang et al. demonstrated that several phytohormones were responded to NPs treatment during the growth and development of plants [52]. It has been described that the biosynthesis of GA was induced during the germination of seeds, while ABA was inhibited [53, 54]. The priming of wheat seeds with various concentrations of iron oxide (Fe_2_O_3_) NPs leads to ameliorated germination potential, enhanced germination uniformity, and significant total germination percentage [55]. Enhanced germination and consistency of the metabolism are attributed to repair all through absorption [56], germination enhancements of metabolites [57], osmotic adjustment and a simple decrease in the reduction of lag time for seeds not dried after treatment [58]. Fast germination occurs due to the synthesis of DNA, RNA and protein during the priming of seed [59]. Plant biomass can also be increased due to synchronized germination and early stand establishment in treated plant seeds [60].

The intrinsic mechanisms of nano-priming-based stimulation of seed germination are still ambiguous. However, few mechanisms related to it were clearly suggested, including the generation of nanopores for increased intake of water, bootstrapping ROS/antioxidant network in seeds, hydroxyl radical’s generation for slackening the cell wall, and stimulant for hastening the breakdown of starch [45]. NP induces ROS when it enters the seed coat and so stimulates various cascades of downstream events [25]. For example, the underlying mechanism of silver (Ag)NPs in the stimulation of seed germination was theoretically suggested, comprising (i) development of nanopores on the seed coat, (ii) gentle ROS stress-persuaded agent, and (iii) acting as a nanocatalyst for elevated starch-hydrolyzing enzyme activity [61]. Hypothetically, seeds soaked in nano-priming solutions (AgNPs) for a limited duration (i.e., 24 h), AgNPs generate OH^−^ radicals which loosen seed coat cell walls and endosperm to induce the germination of seeds [10]. The elevated ROS molecules can stimulate oxidative stress, while at the optimum levels they could be recognized as favorable for the germination of seeds [62].

ROS is essential to break seed dormancy and induce germination, which might be through the induction of GA production and shifting of storage proteins [63]. ROS signaling interacts with GA and ABA, which are linked to seed germination and dormancy [64, 65]. The spatial and temporal production of ROS performs a crucial function in cell-to-cell transmission and the rupture of hydrolytic bonds amidst polysaccharides in the cell wall of seed endosperm [26]. Nano-priming-induced seed dormancy breaking is probably due to the generation of ROS that acts as a positive signal. The aquaporins and ROS play a very important role in triggering the germination of seeds. Earlier reports demonstrated that in addition to intake of water, aquaporins also mediate the diffusion of H_2_O_2_ or ROS beyond biological membranes [66]. Hence, it was hypothesized that the mobility of water into seeds gets shifted after nanopore formation and aquaporin genes were activated, thereby facilitating the dissipation of ROS (H_2_O_2_) via the cell membrane. The increased ROS should be balanced by the seed’s antioxidant network to maintain ROS below the basal level during signal transduction. In view of the linkage between ROS and antioxidant systems, nano-primed seeds are observed with an increased amount of antioxidant enzymes owing to stimulation by AgNPs-based ROS involved in stimulating seed germination [7]. Higher germination was exhibited in the seeds primed with carbon nanoparticles (CNPs) rather than the control seeds [7]. Carboxylic acid-functionalized and stratified CNP’s were set out to be efficient, resulting in improved germination of 90% in green alder (*Alnus viridis* L.) than in control where 60% was observed. Multi-walled carbon nanotubes (MWCNTs) primed Hopbush (*Dodona eaviscosa* L.) seeds evolved with extraordinary seed germination rates and seedling vigor [67]. MWCNTs enable this through elevation of the level of moisture in seeds and activate the increased absorption of water in root tissues [68]. Carboxylic acid-functionalized MWCNT (MWCNT–COOH) was very essential in improving germination of seed membrane lipidome to potentially fix seed dormancy. These observations were found to be compatible with other seeds primed with different NPs in increasing germination rate and seed vigor among different agricultural species [43, 69–71]. Carbon nanotubes (CNTs) act as nano-transporters and can enter cells via cell walls [72]. Moreover, the cylindrical shape of CNTs assists water and gas absorption, thereby keeping seedling germination and its growth at ease in tomato seeds. Interestingly, Aquaporin (*NtPIP1*) gene and its protein (NtPIP1) synthesis were significantly higher in tobacco cells subjected to MWCNTs with reference to control or treated with activated carbon [73]. Furthermore, marker genes like cell division (*CycB*) and cell wall extension (*NtLRX1*) were also upregulated in tobacco cells administered to MWCNTs [73]. The *NtLRX1* gene had been providing support for the cell walls during plant growth and also reacts to external signals [74]. Several studies have indicated that aquaporins act as a crucial part in maintaining plant-water relationships, especially during water absorption, seed germination, cell elongation, regeneration, and photosynthesis [75]. ROS and aquaporins together engage in enhancing the seed germination process, which was confirmed by comparing the nano-primed seeds along with unprimed control and other priming treatments [10]. The increased ROS activates the genes in the aquaporin signaling pathway and imposes modifications to phosphorylation sites in important aquaporin proteins, resulting in enhanced water uptake [76]. ABA regulates crucial aquaporin genes, namely *PIP2, NIP1, TIP3* and *TIP4* during germination [77]. Similarly, in primed seeds, the rapid water uptake is due to the induction of *PIP1* and *PIP2* genes [47].

**Fig. 3:**
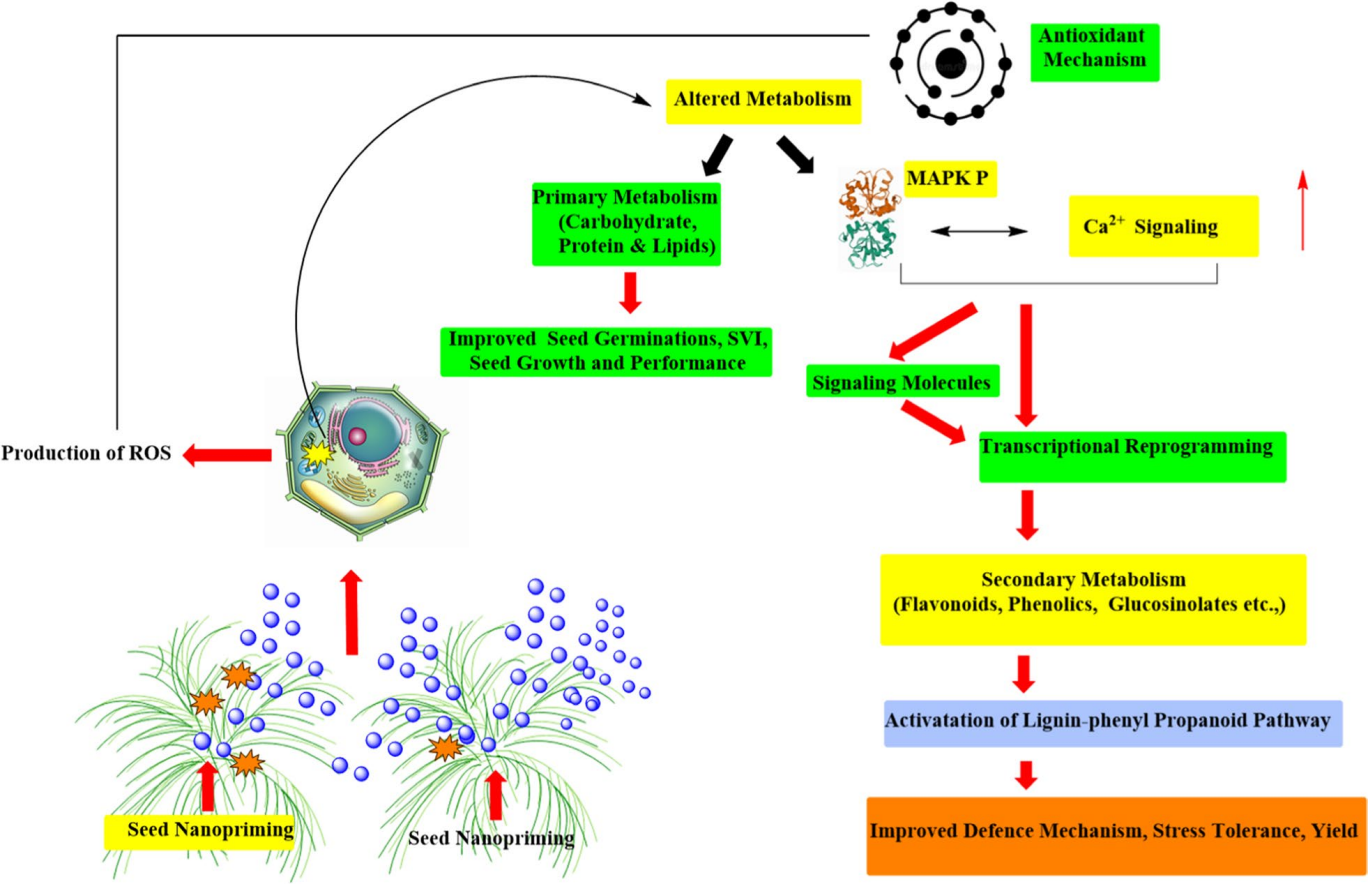
Influence of nano-priming on the primary and secondary metabolism in plants

The induction of metallothionein genes (*MT1* & *MT4*) in tomato seeds treated by nano-priming indicates their probable participation in ROS signaling during germination of NPs treated seeds [48]. A considerably enhanced level of antioxidant enzymes, namely superoxide dismutase (SOD) and catalase (CAT) was observed in nano-primed seeds compared to control. Thus, the greater level of H_2_O_2_ detected in nano-primed seeds could really serve as a signaling agent and was coherent with the concept of oxidative windows, leading to greater germination and hastened seedling growth compared to unprimed and primed seeds [10]. Korishettar et al. concluded that the NPs of Zn and Fe, which have been implemented together with the seed polymer, are capable of entering seeds using the cracks and holes available on the seed coat during the seed imbibition and would enhance enzymatic activity and free radical scavenging by quenching the free radicals, thereby reducing oxidative damage [78]. The dehydrogenase activity as the major cell respiration enzyme, which is higher in nano-primed seed roots than in non-primed control treatments and other processes, can be directly linked to fast water consumption in nano-primed seeds. Higher water consumption can thus improve the germination and development of seeds through complex networks.Moreover, in recent studies, various metal-based NPs such as AgNPs [79, 80], gold (Au)NPs [81], copper (Cu)NPs [82, 83], iron (Fe)NPs [84], iron pyrite (FeS_2_)NPs [85], titanium dioxide (TiO_2_)NPs [86, 87], zinc (Zn)NPs [88], zinc oxide (ZnO)NPs [89–91], and carbon NPs such as fullerene [92] and carbon nanotubes [93] have been utilized used as pre-treatment of seeds for activating germination, growth, and stress tolerance in various crop species. Gold NPs (AuNPs) seed priming often increases water absorption in maize plants [7]. Water consumption for seed germination is extremely important as mature seeds are often dried in nature and require ample water to begin the metabolism and growth of the cells [94]. Sunflower (*Helianthus annuus*) seed germination was improved when the seeds were soaked in nanosilicon solutions at lower concentrations (0.2 and 0.4 mM). The resulting soaked seeds exhibit enhanced germination rate, root length, and SVI [95]. The green synthesized AgNPs (from *Psophocarpus tetragonolobus* (winged bean) leaf extract) seed priming causes a higher germination rate, GVI compared to control and revealed genetic stability [96]. When the seeds of the wheat crop were subjected to silver, copper and Fe NPs, the highest germination percentage was obtained on iron NPs primed seeds. Similarly, the application of Fe NPs stimulated root and shoot growth, whereas exposure to copper NPs significantly reduced it. nano-priming induced seed germination was represented in Fig. 3 [92]. Hence, it was concluded that copper has a repressive action and Fe has an activating effect on wheat seed germination and growth [97].

#### Effect on growth and physiology

**Fig. 4:**
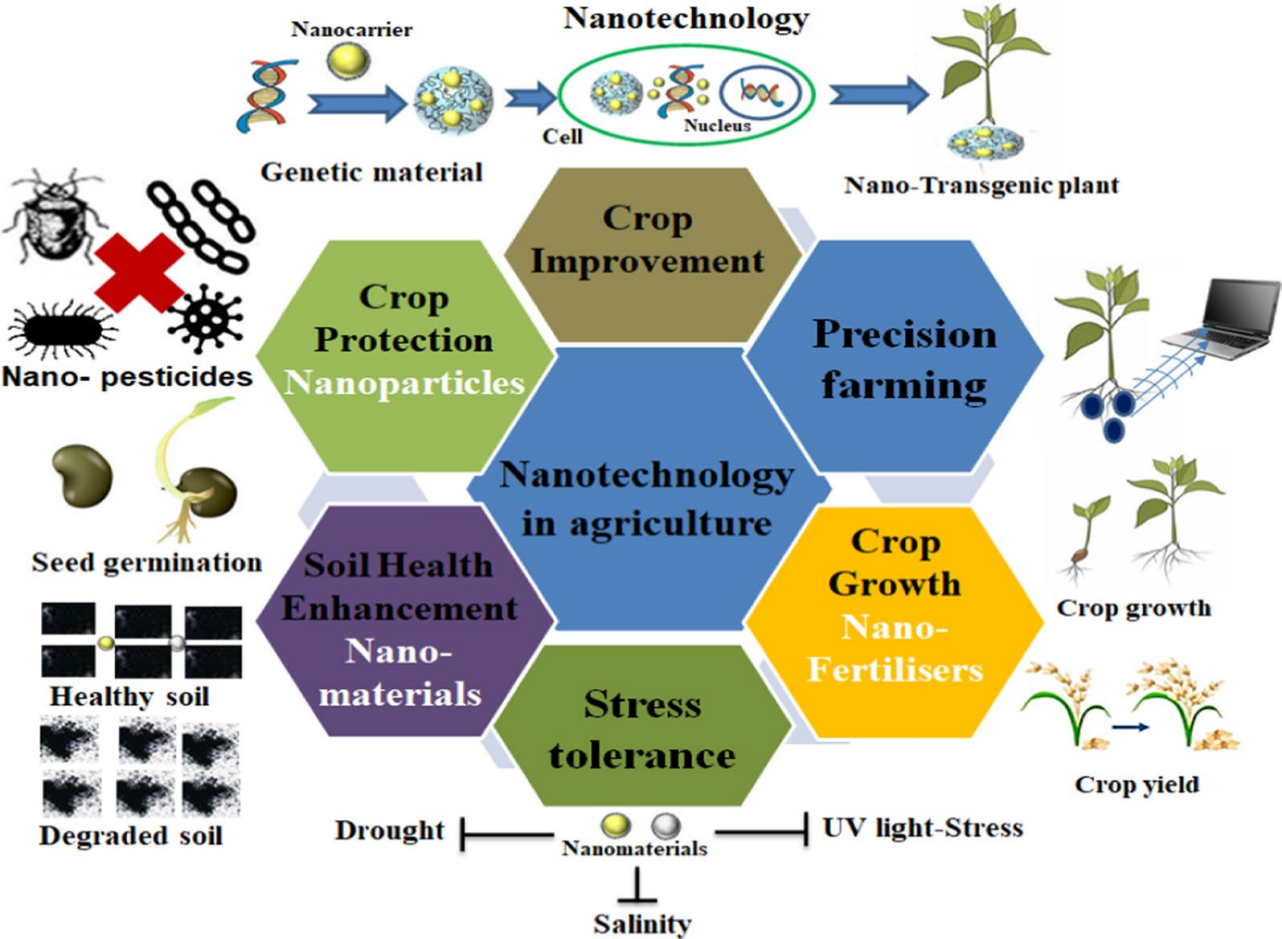
Various applications of nanotechnology in agriculture. This figure was modified and adapted from reference [99] with copyright permission

Nanotechnology is undeniably playing an important role in reviving the agriculture and food production industries [98]. NPs have proved to be a promising alternative to the manufacture of nano-fertilizers compared to conventional fertilizers. Thus, the utilization of nano-fertilizers in agriculture can reduce excess chemical fertilizer usage, thereby controlling environmental pollution [99]. Because of their benefit in enhancing nutritional attributes and tolerance towards stresses in plants, the implementation of NPs was raised. Agricultural nano-formulations were developed by using carriers which can entrap, encapsulate, absorb, or attach active molecules. Different modes of nano-fertilizer applications, such as soil, foliar spray, seed priming, seedling root dip, fertigation drip tape, and aerosol dusting have been employed routinely [99, 100]. Various applications of nanotechnology in agriculture development were described in Fig. 4 [98]. NPs function as a major action in the plant's morphology, growth and physiology [86]. It may precisely or presumptively affect the physiological features through a change in the formation of ROS, peroxidase, SOD, CAT enzyme activities, and modifies the leaf’s protein, chlorophyll, and total phenolic content (TPC) [101]. The large changes in morphological parameters have been distinctly pertinent to the increase in the physiological attributes such as uplift in enzyme activities, greater photosynthetic rate, and significant nitrogen and phosphorus metabolism [102]. Table 1 shows the influence of nano-priming on plant growth and metabolism. Plant health has effectively been determined by a few parameters such as the leaf area, length of shoot and root, and also the root weight. The fresh weight of the root and shoot of wheat was deliberately increased by a lower level of TiO_2_ NPs (10 and 100 ppm, size ~ 20 nm), whereas the same can be decreased with elevated TiO_2_ NPs concentration (> 100 ppm) [146]. There was an improvement in germination rate and root length at low Ag NPs concentrations, which could be attributed to less ROS molecule production [79, 80]. ROS mediates the acceleration of cell cycle entry to G_0_/G_1_ leading to a complementary activity in the plant cell cycle machinery [147]. Meanwhile, when treated with a higher concentration, seedling growth was affected and was believed to be due to the smaller size of synthesized silver NPs [148], which translocate easily to the upper portion of the plant and cause greater toxicity [149]. Also, Navarro et al. confirmed that AgNPs cause toxicity to plants where the cells are affected by their size and the coating [150]. Mahakham et al. detailed a mechanism of nano-priming mediated germination of seeds utilizing biocompatible AgNPs. AgNPs capped with phytochemicals from plant extracts, referred to as nano-priming agents, lead to enhanced seed germination potential via the activation of aquaporin genes with increased ROS generation and accumulation [47]. Previous results suggested that there was an enhancement in the seed percent germination, germination rate index, SVI, fresh biomass, length of radicle and plumules, when lower nano-anatase concentration was used. The most appropriate concentration of nano-anatase was found to be 7.5℅ [151].

**Table 1:** Nano-priming and their impacts on growth and development of different plant species

No.	Crops	Priming NPs	NPs concentration	Physiological/Biochemical/ molecular changes	Refs.
1	*Solanum lycopersicum* L.	Nanosilicon dioxide (nSiO_2_)	8 g/L	Positively affect tomato seed germination	[103]
2	*Capsicum annum* L.	Anatase nanoparticle (nTiO_2_)	nTiO_2_@ 7.5℅	Germination rate index, radicle and plumule length, the fresh weight, and the vigour index were increased significantly	[104]
3	*Eruca sativa*	Polyvinylpyrrolidine-coated AgNPs (PVP-AgNPs)	10 mg/L (PVP-AgNPs)	Increased the root elongation in *E. sativa*	[105]
4	*Citrullus lanatus* (Watermelon)	Silver nanoparticles (AgNPs)	2 mg/mL	Enhanced germination (73.3%) efficiency	[106]
5	*Cucurbita pepo *(Zucchini)	AgNPs	0.5 and 2.5 mg/mL	Induction (86.67% & 90%) of germination rate in zucchini plants	[106]
6	*Vicia faba*	AgNPs	12.5, 25, 50 and 100 mg/L	When compared to control groups, the Ag NPs exposed groups had significantly more chromosomal aberrations, micronuclei, and a lower mitotic index	[107]
7	*Pisum sativum* L. (Pea)	ZnO NPs	125, 250, and 500 mg/kg	Enhanced root elongation	[107]
8	*P. sativum* L. (Pea)	AgNPs	60 ppm AgNPs	Improved seed germination	[108]
9	*Brassica rapa ssp. pekinensis*	AgNPs and AgNO_3_	250 mg/L of AgNPs	Enhanced seed germination and growth rate, chlorophyll content and increased peroxidase (POD) enzyme activity	[80]
10	*Triticum aestivum *(Wheat)	*Phyllanthus emblica fruit extract with* AgNPs (B-AgNPs)	10 mg/L B-AgNPs	B-Ag NPs were beneficial in enhancing early seedling growth, reducing ROS toxicity	[109]
11	Sugarcane (*Saccharum* spp. Cv	AgNPs	25 and 50 mg/L	ROS overproduction overwhelms the antioxidant response of the plant	[110]
12	*Vanilla planifolia* Jacks. ex Andrews	AgNPs	25 and 50 mg/L	i). Effective to eliminate the bacteria without affecting young plants ii). An increase in plantlet antioxidant response, as well as an improvement in nutrient capture	[111]
13	*Nicotiana tabacum*	AuNPs	30, 100 mg/L	Enhancement of growth	[112]
14	Soybean	CeO_2_ NPs	100 mg kg/L CeO_2_ NPs	Improved photosynthetic rate	[113]
15	*Gloriosa superba*	AuNPs	500–1000 μM	Increased seed germination and enhancement of vegetative growth	[114]
16	*Allium cepa*	AuNPs	100, 500, 1000 μM	Acceleration of pollen germination; increase in mitotic index	[115]
17	*Cicer arietinum* L	Fe_2_O_3_ NPs	4 to12 μg/mL	Increased seed germination and growth parameters such as. shoot length, root length	[116]
18	*Pennisetum glaucum*	AuNPs	20–50 mg/L	Promotion of seed germination and root and shoot length	[117]
19	*Arabidopsis thaliana*	AuNPs	10–80 mg/L	Promotion of seed germination	[118]
20	*T. aestivum *(Wheat)	FeNPs	2.0 ppm	Enhances the shoot and root proliferation	[119]
21	*Zea mays*	AuNPs	5–15 ppm	Promotion of seed germination	[47]
22	Buffaloberry (*Shepherdia canadensis* L.) and green alder (*Alnus viridis* L.)	CNPs (MWCNT–COOH)	20 µg/mL and 40 µg /mL	Increased seedling vigor index and germination rate The positive effects on germination and resolution of seed dormancy	[2]
23	*Brassica juncea*	AuNPs	0–400 mg/L	Increased root length	[120]
24	*Z. mays *(Maize)	gold nanoparticles	5–15 mg/L	The maize seeds significantly improved their germination and physiology without any toxicity	[2]
25	*Cicer arietinum* (Chickpea)	TiO_2_ NPs	10–2500 mg/L	Provide protection against cold stress-induced oxidative damage through activation of antioxidant mechanisms in seedlings	[121]
26	*C. annuum* L.	MnNPs	0.1, 0.5, 1 mg/L	The root growth in both non-salt and salt-stressed seedlings significantly improved	[122]
27	*Cucumis sativus *(Cucumber)	CeO_2_ and CuO NPs	(50, 100, and 200 mg/L)	Increased fruit production per plant	[123]
28	*Vicia narbonensis* L. and *Z. mays*	TiO_2_-NPs	0.2, 1.0, 2.0, and 4.0%	In the root tip meristem, there is a large increase in chromosomal aberrations and a decrease in mitotic activity	[124]
29	*Vigna unguiculata subsp. unguiculata* (Black eyed pea plants)	Fe NPs	0.5 g/L	The increased seed weight, leaf chlorophyll and Fe content	[125]
30	*Arachis hypogaea* (Peanut)	nZVI	(40 to 80 μmol/L)	Promote the germination rate of peanut seeds	[126]
31	*Glycine max* (soybean)	CuO NPs	100, 200, and 400 mg/L	In soybean roots, the expression of the *PAL, C4H*, and *CAD* genes were upregulated	[127]
32	*A. cepa L.,* (Onion)	Ag and ZnO NPs	22, 75, and 100 ppm	Impaired cell division, disordered metaphase, chromosomal breaks and cell disintegration in the onion root tips	[128]
33	*T. aestivum* (Wheat)	Ag NPs	10 mg/L	Few proteins related to primary metabolism and cell protection in the shoots and roots exhibit altered expression	[129]
34	*Oryza sativa* (Rice)	CeO_2_ NPs	125 mg/L	Lipid peroxidation were significantly increased in the rice roots	[130]
35	*S. lycopersicum* (Tomato)	NiO NPs	2.0 g/L	An elevated CAT, GR, and SOD activities in tomato plants upon NiO NPs exposure	[131]
36	*Brassica juncea *(Indian mustard)	CuO NPs	500 mg/L	SOD activity in roots and shoots was substantially increased	[132]
37	*B. Juncea*	Gold NPs	10 and 25 mg/L	Marked increase in the chlorophyll contents	[133]
38	*Asparagus officinalis*	AgNPs	100 mg/L	Increased ascorbic acid and chlorophyll contents	[134]
39	*S. lycopersicum *(Tomato)	(SWCNHs)	25 µg/mL	Improved germination rate	[135]
40	*Crocus sativus*	AgNPs	40, 80, 120 ppm	Improved root growth from blocking of ethylene and classical stress signaling reactions (mediated by [Ca^2+^] cyt and ROS) and a specific effect on the plasma membrane conductance	[136]
41	*Lycopersicum esculentum*	Nano-silicon oxide	8 g/L	Improved seed germination	[103]
42	*Hordeum vulgare* seeds	FeO NPs (Nano-zero-valent iron (nZVI)	250 mg/L	Increased root length	[137]
43	*Solanum tuberosum* (Potato)	AgNPs	150 ppm	Improves the chlorophyll content and can equally enhance catalase activity	[138]
44	*T. aestivum*	AgNPs	0.01–1.0 mg/L	Promotion of respiration intensity, seed vigor, and seed germination; increase in dry biomass of roots and aerial parts	[139]
45	*A. thaliana*	AgNPs	0.01–100 mg/L	Increase in root length, biomass, and evapotranspiration	[140]
46	*B. juncea*	AuNPs	10 and 25 ppm	The improved concentration of chlorophyll contents and faster rate of CO_2_ fixation in the photosynthetic phase, lead to higher soluble sugars	[133]
47	Tomato	CoFe_2_O_4_ NPs	Up to 1000 mg/L	Maintaining the chlorophyll contents in tomato leaves	[141]
48	*O. sativa* L. cv. (KDML 105)	AgNPs	5 and 10 ppm	Up-regulation of aquaporin genes for enhancing seed germination	[2]
			10 and 20 mg/L	Induced water uptake level	
49	*S. lycopersicum *(Tomato) *and A. thaliana*	Carbon nanotubes (CNTs) and AgNPs	(CNTs) (40 μg/mL) and AgNPs (0.2 and 0.5 mg/L)	Significantly activate the expression of aquaporin genes in tomato roots and *Arabidopsis* seedlings	[142]
50	A.*thaliana*	AgNPs	0.2 or 0.5 mg/L	Transcript levels of aquaporins such as *PIP 1;2, PIP 2;1, PIP 2;2, SIP 1;1*, and *TIP 1;1* increased approximately twofold over control plants	[143]
51	*Spinacia oleracea *(Spinach seeds)	FeS_2_ NPs	FeS_2_ (80 mg/mL of water)	FeS_2_ NPs could enhance the amylase enzymatic activity in spinach seeds	[85]
52	A.*thaliana*	AgNPs	0–100 μM	i). Increase in root length ii). Activation of expression of genes implicated in cell proliferation and metabolism; activation of expression of hormonal signaling related genes	[140]
53	*S. lycopersicum *(Tomato)	CNTs	(10–40 g/mL)	The alteration of seed membrane, increased rate of germination and plant growth	[144]
54	*S. lycopersicum *(Tomato)	Carbon nanotubes (CNTs)s	10–40 mg/L	The dramatically increase germination rate and enhanced growth of tomato seedlings with up-regulation of the aquaporin (water channel gene)	[145]
55	*Zea mays* L. *(Maize-* waxy variety)	GNPs (Galanga rhizome extracts (GRE) 2 mL GRE: 10 mL HAuCl_4_)	GNPs at 5 mg/L 10 mg/L GNPs at 15 mg/L	Increased total chlorophyll (35–53%) contents in all GNP priming treatments as compared to unprimed plants	[47]
56	Jasmine rice (*O. sativa* L. cv. KDML105)	AgNPs	10, 20 mg/L	Seedlings in the Ag NPs10 and Ag NPs20 priming treatments had 2.6 and 2.5 times higher α-amylase activity than control seedlings. Catalase activity increased by 71% and 61% in primed seeds after 24 h of imbibition, respectively	[47]

Laware and Raskar published in their research paper that a low concentration of TiO_2_ NPs upgraded the seed germination percentage and seedling growth rate with a synchronous elevation in the amylase and protease activities [151]. In oriental lilies, AgNPs enhanced the content of potassium (K) while decreasing the content of magnesium (Mg), phosphorus (P), and sulphur (S) [152]. At the plant level, these mechanisms may comprise genotoxicity, plant-related variations in the uptake of minerals, production of ROS, which suppresses photosynthesis, and the exchange of gases, and may lead to reduced plant growth and biomass. When MWCNTs were used, there was a positive response recorded towards the plant growth against the untreated crops [153]. Single-walled carbon nanohorns (SWCNHs) at its highest concentration (100 µg/mL) made all crops germinated except tomato. However, there was the greatest germination rate of the tomato crop at its lowest concentration (25 µg/mL) of the nanomaterials [135]. MWCNTs acting as a nano-fertilizer not only initiate the growth and development of plants, but also promote photosynthesis, induce aquaporin expression, antioxidant defense, and supplement nutrition [154]. Nanomaterials supplemented on pepper leaves could act as a fertilizer such as the foliar sprayed CuO NPs, ZnO NPs, MgH NPs and MgO NPs at a proper concentration was found to increase the chlorophyll content, plant’s height and leaves growth more than the control [155].

ZnO NPs foliar spray with a limited concentration of 10 mg/L remarkably increased the plant biomass, shoots and root length, and root area of cluster bean. In addition, they appreciably boosted the contents of chlorophyll (276.2%), whole soluble leaf protein (27.1%), and activities of various enzymes like acid phosphatase (73.5%), alkaline phosphatase (48.7%), and phytase (72.4%) as compared to control. The total lipids, proteins, amino acids, thiols, and chlorophyll concentrations were significantly increased after being treated with varying concentrations of sulfur and zinc oxide NPs as compared to the untreated control [156]. It has also been shown that better seed germination, root-shoot length, pigment content, improved total antioxidant activities (TAA), 2,2-Diphenyl-1-picrylhydrazyl (DPPH) and flavonoid content, and decreased lipid peroxidation were observed after nano zinc sulfide (nZnS) treatment [157]. Rai-Kalal and Jajoo investigated the beneficial impacts of seed priming with ZnO NPs in wheat cultivar H–I 1544 and observed that wheat seeds primed with ZnO NPs (10 mg/L) had a significant positive impact on seed germination efficiency and seed vigour index (calculated by multiplying germination (%) and seedling length) when compared to unprimed (control) and hydro primed seeds [158]. Yuan et al. studied the cellular response of *Capsicum annuum* to Fe NPs [159]. Lower concentrations of Fe NPs resulted in significant plant growth compared to high concentrations. This is likely due to the aggregation of most iron NPs in the cell walls, which shifted further into roots via the apoplastic (non-living) pathway. While at a low or optimal level, NPs contribute to controlling vascular bundle development, increasing chloroplast numbers, and grana stacking. After absorption of iron NPs in the roots were further mobilized to the leaves and stems in available patterns to support the growth of the plants. Iron oxide NPs priming in chickpea improved germination significantly along with enhanced the level of lipophilic non-enzymatic antioxidants [160]. The nanoformulation of fertilizers (nano iron chelate and nano zinc) enhanced the phosphorous levels, biomass, crude protein and soluble sugar contents in *Zea mays* L as compared to chemical types of fertilizers [161].

Bio-priming of seeds with plant growth-promoting rhizomicrobes (PGPRMs), like nano-priming, is an important approach in sustainable agriculture [162, 163], particularly in vegetable plantations [164]. Bio-nano-priming is a convergent field of applied technology that aims to enjoy the combined benefits of nano and bio-agent application growth of agricultural plants, helping in enhanced crop cultivation [165]. Nanoparticles revealed three modes of association with crops, PGPRMs and the soil elements. When PGPRMs are treated with metal or metal oxide NPs, they might cause changes in their profiles of the synthesis of secondary metabolites and proteins in a concentration-based manner [166]. The application of metal/metal oxide or carbon NPs at a minimal level to broth cultures of microbial cells improved the production of plant hormones (IAA) [167] and siderophores (metal-chelating ligands) [168, 169] that may fine-tune the plant growth-promoting benefits in treated crops through co-immobilization/coating of the crop seed [170]. The approach to bionano-priming might comprise coadministration of NPs and PGPRMs on seed [171], application of immobilization of helpful PGPRMs on nanomaterial matrix to the surface of subjected seeds [171], administration of PGPRMs or its bioactive compounds, namely microbial cyclo-peptides, on the seed surface [172], followed by exposure of the NPs through foliar or seedling roots [173], and coating seeds using NPs primed PGPRMs [174]. Hence, this strategy can be useful for enhanced seed germination, SVI and plant growth.

### Influence of Nano-priming in primary metabolism

**Fig. 5:**
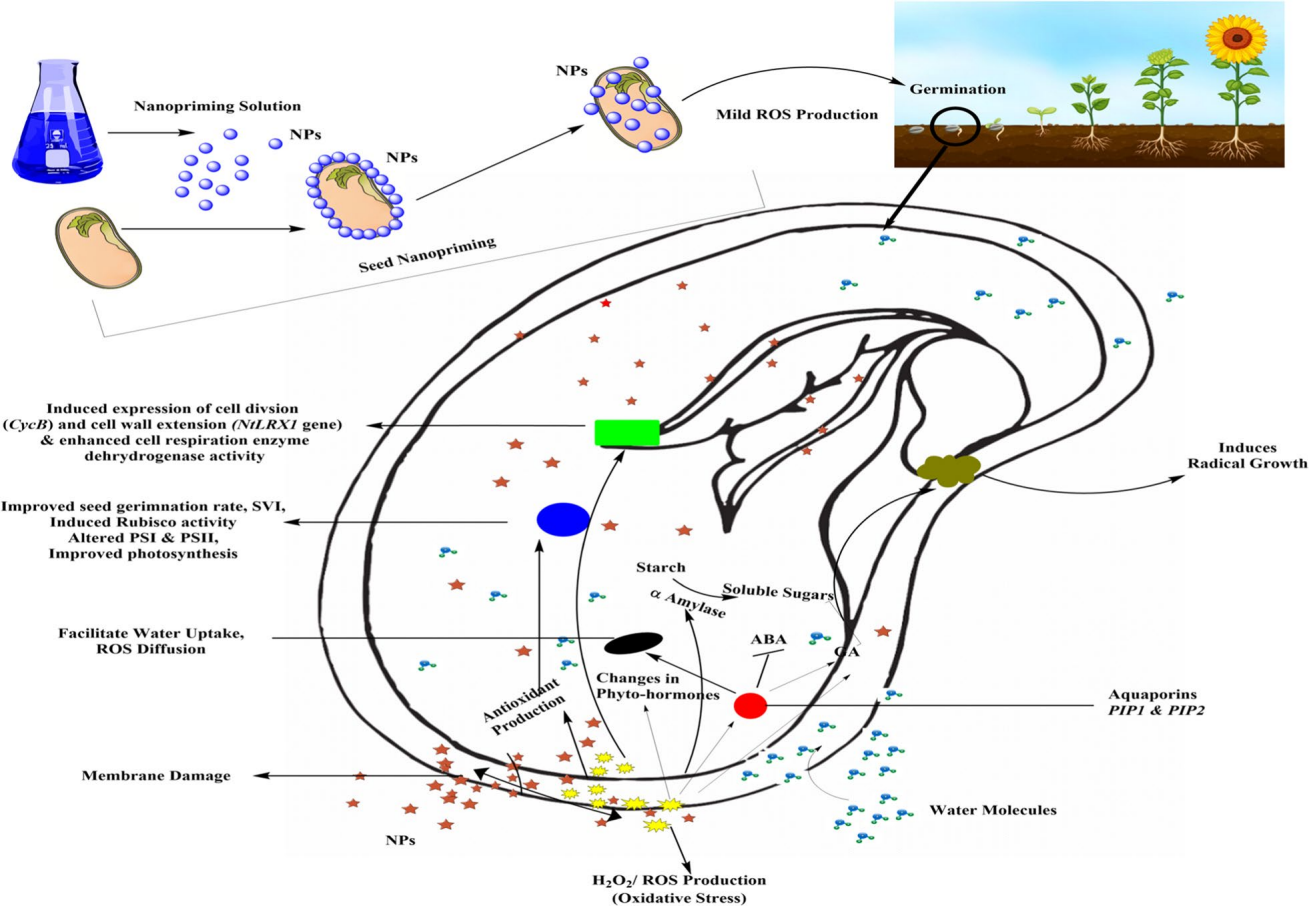
Influence of seed nano-priming on the seed germination potential and plant growth

Primary metabolic pathways are crucial for a plant’s life, and the metabolites synthesized are directly involved in plant growth (photosynthesis) and metabolism. The impact of nanoparticles on plant metabolism was shown in Fig. 5 [175]. Fullerenol nano-priming in wheat boosted primary metabolism to increase growth and productivity under salt stress, as evidenced by the concentration-dependent drop in phenolics and flavonoids content [176].

#### Impact on photosynthesis

Photosynthesis is the process through which higher plants, algae, and certain microorganisms convert sunlight energy into energy-rich organic compounds and store it. Nano-priming has derived its various outcomes from the plant’s photosynthesis mechanism [5]. Silicon dioxide (SiO_2_) NPsboost the synthesis of photosynthetic pigments and carbonic anhydrase activity, ultimately improving the photosynthetic rate [103]. Siddiqui et al. found that SiO_2_ NPs in *Cucurbita pepo* leaves, not only upgraded the photosynthetic competence but also improved plant defense activity to counter saline stress by increasing chlorophyll content, water capacity, net photosynthetic rate, amount of transpiration, proline and carbonic anhydrase, and stomatal conductance [103]. The priming of Egyptian roselle (*Hibiscus sabdariffa* L.) cultivars with a lower concentration (0.01%) of aluminum oxide (Al_2_O_3_) NPs) significantly improved growth features (leaf area, length of shoot, root, fresh and dry mass), as well as physiological and biochemical functions (carotenoid level, chlorophyll a, chlorophyll b, soluble sugars, protein, proline), and the functions of various antioxidant enzymes such as SOD, CAT, peroxidase (POD), and ascorbate peroxidase (APX). Thus, seed priming in *Egyptian roselle* (*H. sabdariffa* L.) cultivar could stimulate positive impacts at lower concentrations [177]. Pradhan et al. described that manganese (Mn) NPs upgraded the photosynthetic rate by mediating water splitting in the electron transport system [178]. During photosynthesis, a crucial enzyme called Rubisco (Ribulose-1, 5-bisphosphate carboxylase/oxygenase) is essential to catalyze and integrate carbon dioxide into biological compounds. In chickpea, TiO_2_ NPs stimulated the photosynthesis process and the Rubisco enzyme activity [179]. Samadi et al. showed that TiO_2_ NPs (200 mg/L) employed on *Mentha* sp. manifested a positive response on chlorophyll a and b and carotenoid contents [180]. Similar observations by Yang et al. showed that TiO_2_ NPs improved photosynthesis by stimulating light absorption in the chloroplast, normalizing the provision of light energy from photosystem I to photosystem II, oxygen evolution, water splitting, and raising Rubisco enzyme activity [181].

Iron-based NPs play a pivotal function in electron transport while respiration and photosynthesis. These are transported to the leaves to help photosynthesis, the reproductive organs [182] and to the seeds to initiate embryogenesis [183]. As described by Rui et al. [184], Fe_3_O_4_ NPs can be an iron-rich origin for *Arachis hypogaea* plants, substituting typical regular sources of iron. The supplementation of Fe_3_O_4_ NPs improved plant height, root length, chlorophyll and Fe levels, and modulated the activity of antioxidant enzymes and phytohormones (diminished ABA and enhanced GA_3_ content). Seed priming with low zero-valent iron nanoparticles (nZVI) concentration (< 80 mg/L) increased the photosynthetic pigments in rice seedlings and favorably induced the growth of the rice seedlings. Chlorophyll a/b ratio revealed no aberrant alterations in control and nano-primed seedlings designated that no stress was stimulated owing to the administration of nZVI at a lower concentration [6]. When rice (*Oryza sativa* L. cv. Gobindobhog) seeds were primed for 72 h with 20 mg/L nZVI, several biochemical and physiological changes were observed at various time points (5, 10, 20, 40, 60, and 80 h). Apart from that significant up-regulation of genes like *OsGA3Ox2*, *OsGAMYB*, which are responsible for controlling the activity of many hydrolases and mediating the efficient mobilization of seed storage food reserves, was noticed in both the nZVI and the diphenyleneiodonium (DPI) and nZVI co-primed sets. In wheat leaves, photosynthetic pigment content was found to be higher after aluminum NPs treatment [185]. Similarly, cesium oxide NPs not only promoted the transpiration rate and stomatal conductance in kidney beans [186] but also increased the activity of Rubisco in soybean [113]. In the mung bean leaves, gold NPs intensified the rates of electron transport and oxygen evolution in chloroplasts [187].

#### Starch metabolism

α- Amylase is a hydrolytic enzyme which degrades the reserved carbohydrates to soluble sugars in order to sustain an active respiratory metabolism that aids in the germination of seeds and plant growth prior to photosynthesis [188]. Increased α-amylase activity, which causes rapid starch degradation in germinating seeds of nano-priming treatment, may have an indirect effect on increased germination rate and seedling vigor [47]. A high sugar concentration in the cells reduces osmotic potential and water potential, triggering seedling growth and accelerating germination [47]. Man et al. observed that the surface of starch granules was initially pitched by α-amylase followed by protruding into inside and the granule from the inside out, suggesting an increased stimulation of the activity of α-amylase in the seeds treated by the nano-priming technique [189]. Moreover, this stimulation of α-amylase production is reliant on GA activity. For instance, Mahakham et al. reported that the absence of α-amylase biosynthesis is linked to the lack of GA [47]. This indicates that α-amylase induction is reliant on GA activity and reveals signaling crosstalk present among NPs, α-amylase and GA in nano-primed seeds. While the upstream GA signaling factors involved in α-amylase-mediated starch hydrolysis are unknown. It should be interesting to explore the crosstalk between plant hormones and sugar signaling cues sponsored by nano-priming. AgNPs were usually coated with phytoconstituents such as the plant extract of kaffir lime. Phytosynthesized AgNPs primed on old rice seeds at a concentration of 5–10 ppm considerably revamped germination and seedling vigor with reference to unprimed, AgNO_3_, and traditional hydropriming rice seeds [47]. Furthermore, the activities of α-amylase and total soluble sugars were also determined in seedlings of rice for examination of starch metabolism. Notably, a remarkable increase in α-amylase activity was observed after 6 days of germination in nano-primed rice seedlings compared to unprimed and other primed seedlings. Nano-priming not only increased α-amylase activity (greater soluble sugar facilitates seedling growth) but also up-regulates aquaporin genes in germinating seeds [47]. AgNPs primed seeds could induce gentle oxidative stress, triggering the germination of seeds through loosening of the cell wall. The ability of AgNPs to penetrate the seeds might behave as a nanocatalyst in the enzymatic digestion by α-amylase and hence rapidly magnify the rate of the reaction [47]. Starch hydrolysis occurring at a faster rate was believed to occur due to a change in the α-amylase conformation, but the active site of the enzyme retained its catalytic function [190]. As a result of the enzymes conformational change, the enzyme activity and stability were intensified leading to greater starch degradation, and this may be associated with the interaction of molecules overlaid onto the coverings of silver nanocarriers. Furthermore, the induced α-amylase could feasibly interplay with functional groups present on the coverings of phytochemicals-overlaid AgNPs forming thiol linkages and performing catalysis [47]. Like AgNPs, it was proposed that FeS_2_ NPs could mimic amylase activity to boost starch degradation in spinach seeds [191]. Nano-priming with nZVI also remarkably boosted amylase and protease activity during germination, thereby leading to early radicle emergence [25]. Laware and Raskar outlined those lower levels of TiO_2_ NPs upgraded the seed germination and seedling growth rate while simultaneously increasing the amylase and protease enzyme activity [151]. Correspondingly, Yang et al. revealed that TiO_2_ NPs are involved in water absorption, thus improving seed germination and beneficial in boosting their photosynthetic efficiency and nitrogen metabolism [181]. The chemo blended AgNPs treated wheat exhibits reduced proteins related to carbohydrate metabolism (glycolysis), redox, and mitochondrial electron transport chain [192] while enhancing the proteins associated with secondary metabolism and increasing the enzymes of antioxidant machinery like SOD, CAT, and peroxidase [193]. Proteins related to glycolysis were decreased in soybean upon exposure to AgNPs [194], while enhanced with Fe and CuNPs [195]. Fe NPs increases the proteins associated with photosynthesis and protein metabolism [196].

### Role of nano priming in lipid metabolism in overcoming seed dormancy

A relatively few studies have shown cell membrane lipid modifications can contribute to alleviating seed dormancy in crops. Seed dormancy in agricultural crop species could be aided by lipid modification in the cell membrane [197, 198]. At the time of germination, membrane lipids on the seeds were noted to be reorganized by the steady-state increase of plastidic lipids, where the content of PA (phosphatidic acid) initially diminished and gradually increased, followed by a final decrease in soybeans [199]. Fatty acid variation with respect to embryo dormancy and a distinctive alteration in linoleic acid contents in the seeds of *Amaranthus albus* before and after cracking dormancy was also noticed [200]. The modulation of lipid metabolism in plants was recently shown to be induced by NPs [52, 99]. The roles of lipid metabolism in overcoming seed dormancy the membrane lipids in upland boreal forest plant species (buffaloberry (*Schepherdia canadensis* L.) and green alder (*Alnus viridis* L.) were examined to see if seed membrane lipid metabolism plays a role in the release of dormancy in seeds after priming with NPs.

In each of these upland species, roughly 12 membrane lipid classes and related molecular species have been determined quantitatively. These comprise lysophosphatidylcholine (LPC), cardiolipin (CL), PA, lysophosphatidylethanolamine (LPE), phosphatidylethanolamine (PE), phosphatidylcholine (PC), phosphatidylinositol (PI), sulfoquinovosyl diacylglycerols (SQDG), phosphatidylglycerol (PG), digalactosyldiacylglycerol (DGDG), phosphatidylserine (PS), and monogalactosyldiglyceride (MGDG) [197]. Green alder seeds (*A. viridis* L.) primed and layered using MWCNT–COOH result in improved germination rate (90%) and seed membrane lipidome to effectively reconcile seed dormancy [198]. The reconfiguration of C18:3 enriched fatty acids in the seed membrane lipid moieties: PG16:1/18:3, PC18:1/18:3, PE18:3/18:2, and DGDG18:3/18:3, which corresponds to an increase in germination, seedling vigor, and releasing the dormancy of both embryo and seed coat in upland boreal forest species. Precisely, the biosynthetic avenues including PA, DG, PC, and PE seem to be regulated by MWCNT–COOH, easing seed coat and embryo dormancy, thereby improving germination and SVI in upland boreal forest species [197]. Martinez-Ballesta et al. [201] showed that nano-priming using carbon NPs (CNPs) in broccoli upgrades the aquaporins, ions, and water mobilization in cell membranes. CNPs assisted in balancing electrostatic interactions in cell membranes and the membrane developed new lipid domains, or rafts, as an outcome of the CNPs activating the lipid metabolism.

### Modulation of plant secondary metabolism by NPs

Several previous studies have indicated that ROS-induced signaling events play a pivotal part in the activation of secondary metabolism [202]. Moreover, the generated ROS acts as a signal for other messengers such as jasmonic acid (JA) [203], salicylic acid (SA) [204], ethylene (ET) [205], nitric oxide (NO) [206], and brassinosteroids (BRs) [207], which has the ability to regulate secondary metabolisms directly or indirectly [101]. It is believed that the NP-induced responses include enhanced ROS, cytoplasmic Ca^2+^ and induction of mitogen-activated protein kinase (MAPK) cascades that resemble other abiotic stresses [208]. To support this notion, it was also reported that genes related to ROS pathways and ion homeostasis suggesting that ROS and conserved Ca^2+^ transduction cascades have been found to be expressed in the root transcripts of cotton seeds primed with poly (acrylic acid) coated CeO NPs under salt stress [209]. AgNPs induce Ca^2+^ bursts and ROS induction when binding to plasma membrane-bound receptors in *Arabidopsis* [210]. Proteomic studies of AgNPs treated rice roots also revealed the presence of Ca^2+^ levels and associated signaling pathway proteins [211]. The hypothesis of the above studies also indicated that AgNPs or ions liberated, interfere with cell metabolism by affixing to Ca^2+^ receptors, Ca^2+^ channels, and Ca^2+^/Na^+^ ATPases. As recognized by calcium-binding proteins (CaBPs) or other NP-specific proteins, NPs either resemble Ca^2+^or signaling molecules in the cytosol [176]. Phosphorylation of MAPKs and induction of downstream TFs result in the transcriptional reprogramming of the metabolism of secondary metabolites in plants [212]. There is no direct evidence for the involvement of MAPK pathways in plant-NP interactions is available. However, it was hypothesized that when exposed to AgNPs, plants would most likely use the MAPK cascade [213].

#### NPs induced secondary metabolites production

Plants may be protected by a process that enhances their bioactive compounds to resolve metal toxicity under abiotic stress conditions [214]. Gene expression analysis is a powerful approach to understanding the molecular mechanisms underlying reactions in plants exposed to nanomaterials. Biosynthetic genes for flavonoid and anthocyanin in *Arabidopsis* are up-regulated upon the exposure of AgNPs [215]. Anthocyanin pigment 1 (*PAP1*) and anthocyanin synthase 1 (*ANS1*) genes have been implicated in anthocyanin biosynthesis, which is frequently synthesized during abiotic stresses. The levels of *PAP1* and *ANS* transcripts were slowly increased at elevated concentrations (5.0 and 10.0 mg/L) of AgNPs treated turnip seedlings, which also correlated with a higher accumulation of anthocyanin [79]. *PAP1* and *ANS1* expression in Chinese cabbage seedlings was gradually increased from low to high concentrations of AgNPs (250 and 500 mg/L) [80]. Similarly, the production of anthocyanin and the expression of their pathway genes upon AgNPs treatment suggest that AgNPs could activate the biosynthesis of anthocyanin in Chinese cabbage seedlings [80].

The phenylalanine ammonia-lyase (PAL) and chalcone synthase (CHS) enzymes, as key enzymes in the production of phenylpropanoid compounds, serve a prime role in plant responses to a/biotic stresses [216]. It is noted that in numerous plants, environmental stresses have been shown to affect the expression of the *PAL* gene [217]. The expression of *PAL* and *CHS* were significantly induced by the AgNPs (40 ppm) treatment in *Portulaca oleracea* seedlings compared to control [216]. *PAL* expression was significantly induced by AgNPs in turnip [79] and Chinese cabbage seedlings [80]. In comparison to unprimed and hydroprimed controls, Kumar et al. identified that winged bean seeds (*Psophocarpus tetragonolobus* L.) primed with 50 mg/L AgNPs significantly improved growth performance (GP) by 53.64 percent and 31.54 percent, respectively, and SVI by 68.24% and 57.59% [96]. The flavonoid and phenolic biosynthesis pathway genes such as *PAP1*, *ANS*, *PAL,* and flavonol synthase (*FLS*) displayed enhanced expression in *B. rapa* ssp. *rapa* seedlings upon priming with CuO NPs [101]. In association with the gene expression, the CuO NPs (500 mg/L) treated turnip seedlings accumulated the highest total phenolic and flavonoid content in *B. rapa* ssp. *rapa* seedlings [101]. CuO NP (500 mg/L) treated *B. rapa* ssp. *rapa* seedlings had significantly higher levels of hydroxybenzoic acid (604.43 g/g), hydroxycinnamic acid (720.78 g/g), and flavonol (1344.49 g/g) than untreated seedlings (390.68, 490.86, and 828.42 g/g, respectively [101]. Similarly, NiO NPs (250 and 500 mg/L) were shown to induce the expression of phenolic biosynthesis genes like *PAP1, ANS,* and *PAL* in Chinese cabbage seedlings [218]. NiO NPs (500 mg/L) treated Chinese cabbage seedlings exhibit notably elevated hydroxybenzoic acid (921.65 μg/g), hydroxycinnamic acid (890.38 μg/g), and flavonol (1228.18 μg/g) concentrations compared to untreated plants, at 659.41, 706.62, and 659.41 μg/g, respectively. Similarly, TPC and total flavonoid content (TFC) were higher in NiO NPs (500 mg/L) administered seedlings than in control plants [218]. AgNPs (20 & 50 ppm) treated seedlings of *Echium amoenum* revealed a considerable increase in total phenolic content compared to the untreated seedlings [219]. The application of silver (Ag) and platinum (Pt) NPs enhanced the TPC level by 17 and 15%, respectively, compared to the control seedling of lettuce [220].

Upon exposure to the least concentration of AgNPs (1.0 mg/L) in *O. sativa* enhanced the carotenoid biosynthesis-related genes (*CYB* and *ZEP1*) [221]. While higher concentration of AgNPs inhibits the carotenoid production in the rice, turnip and Chinese cabbage seedlings [79, 80, 221]. Yue et al. have shown that lignin biosynthesis genes *ZmPAL*, *ZmCCR2* (Cinnamoyl-CoA reductase) and Z*mCAD6* (Cinnamyl alcohol dehydrogenase) have been up-regulated in maize roots exposed to lanthanum oxide NPs (La_2_O_3_ NPs) [222]. Wang et al. stated a notable decrease in the chlorophyll synthesis genes such as *Chlorophyll an oxygenase* (*CAO*), *Chlorophyll synthase* (*CHLG*), *Copper response defect 1* (*CRD1*), *Magnesium-protoporphyrinix methyltransferase* (*CHLM*), and *Mg-chelatase subunit D* (*CHLD*) in ZnO NPs treated *Arabidopsis* plants [223]. Expression of carotenoid synthesis genes, primarily ZDS, has increased profoundly in ZnO NP supplemented plants [223]. The level of chlorophyll a and b declined by > 50%, whereas the level of carotenoids remained hugely uninfluenced in *Arabidopsis* plants treated with 300 mg/L ZnO NPs [223]. The exposure to zinc oxide and selenium NPs profoundly stimulated the expression of rosmarinic acid synthase (*RAS*) and hydroxyphenyl pyruvate reductase (*HPPR*) genes in *Melissa officinalis* [224]. El-Badri et al. used SeNPs and ZnO NPs during seed imbibition and the early seedling stage in two rapeseed cultivars to investigate the effect of nano-priming on plant hormones and germination processes during salinity stress. Nano-treatment increased the chlorophyll a, b, and total chlorophyll content, as well as the final germination percentage, germination rate, seed microstructure, and antioxidant activity [225]. Genes related to glucosinolate (GSL) biosynthesis and regulation (*BrMYB28, BrMYB29, BrMYB34, BrMYB51, St5C* and *SUR1*) have been increased in seedlings treated with AgNPs (5 and 10 mg/L) and (250 and 500 mg/L) respectively in turnip and Chinese cabbage young plants [79, 80]. Zhang et al. reported that the GSL synthesis-based plant defense pathway might be one of AgNPs exposure hallmarks [226]. The expression of aliphatic GSL (*BrMYB29* and *BrMYB28*) and indolic GSL regulatory genes (*BrMYB51* and *BrMYB34*) were upregulated in CuO NPs (250 and 500 mg/L) treated turnip young plants [82]. Similarly, CuONPs (500 mg/L) exposure to young plants has considerably improved the content of aromatic, indolic and aliphatic GSLs in *B. rapa* ssp. *rapa* [82]. The exposure of Chinese cabbage young plants to nickel oxide nanoparticles (250 and 500 mg/L), in turn, upregulates the GSL TF genes namely *BrMYB28, BrMYB29, BrMYB51,* and *BrMYB34* [218]. UHPLC-TQ-MS revealed five aliphatic glucosinolates (glucoallysin, gluconapin, progoitrin, glucobrassicanapin, and sinigrin), four indolic glucosinolates (4-hydroxyglucobrassicin, 4-methoxyglucobrassicin, glucobrassicin and neoglucobrassicin), and one aromatic glucosinolate of gluconasturtiin. Aliphatic, indolic and aromatic glucosinolates were expressively amplified in the nickel oxide nanoparticles (500 mg/L) exposed Chinese cabbage seedlings [218]. In addition to nano-priming, the application of nanomaterials was used to induce the production of phytochemicals in plant cell cultures, cell lines, callus cultures, and hairy root cultures (HRCs). AgNPs (900 mg/L) treated in *Artemisia annua* HRCs lead to the induction of artemisinin production by up to 3.9 folds along with higher oxidative stress, MDA generation, and CAT activity [227]. Fenugreek treated with AgNP (2 mg/kg) leads to significant plant growth and diosgenin accumulation [228]. Anthocyanins and the genes related to flavonoid biosynthesis were increased in AgNPs treated *A. thaliana* [215]. Cadmium oxide (CdO)NPs-treated barley plants showed enhanced production of ferulic acid and isovitexin [229].

### Cytotoxic and genotoxic responses of Nano-priming

As a result, the nanoparticles have both negative and positive effects on seed germination, root elongation, cell division, growth, and metabolic functions as a result obtained from various phytotoxicity reports [79, 80, 221]. During germination, various processes take place, such as the transcription of the gene and its translation, repair of DNA, breathing and energy metabolism, respectively [59]. The rate of root growth appears to concur with the mitotic index and the latter reflects the frequency of cell division [230]. Micronuclei formation has been considered a real mutagenic effect, leading to damage of genetic material and malformation of spindle fibers as the result of DNA double-strand breaks [231]. Plants exposed to Ag, Cu, TiO_2_, Zn, ZnO, SeO, MWCNTs, tetra-methyl-ammonium-hydroxide (TMAH) and bismuth oxide (Bi_2_O_3_) NPs create the most apparent anomalies and oddities such as micronuclei forming, perturbed chromosomes, fragmentation of chromosomes, stickiness, bridge, laggards’ chromosomes and reduction in a mitotic index. The difference in concentration, nanoparticle size and duration of exposure leads to a difference in severity in terms of their abnormalities. The concentration of NPs used for priming studies determines the cytotoxic and genotoxic effects and it varies between plant species as well. According to Shang et al., NPs with diameters varying from 8 to 10 nm can enter the nucleus via nuclear pores, indicating a size-dependent interface of nanoparticles with cell constituents [232]. The NPs might also disrupt cell cycle checkpoints, come into contact with antioxidant enzymes, or stimulate ROS generation by cellular constituents through mechanical or chemical bonding to proteins, resulting in protein inhibitory and defects in cell division mechanisms. Interruptions or reductions in DNA repair function, as well as an increase in oxidative stress caused by ROS produced during interplay with cell organelles (cell membrane, mitochondria), result in antioxidant reduction and changed gene expression [233]. The toxicity of NPs is caused by three distinct mechanisms [234]. Firstly, the toxic materials from NPs are released into the contact media. For example, free Ag^+^ions discharged from AgNPs or other toxic ions produced from soluble nanoparticles might also lead to DNA injury. Ag^+^, Cu^+^, Fe_2_^+^, Cr_5_^+^Ni_2_^+^, and Mn_2_^+^ as transition metal ions are involved in ROS induction and formation via Fenton-type reaction and these ions could even bind to DNA base pairs as well [235].

Many researchers have reported on mammalian and bacterial cytotoxicity, while the role of NPs in contributing genotoxicity in plants has received little attention. AgNPs have been found to cause cytological changes in the root tips of germinated wheat and barley seeds [129]. Silver nanoparticles seed pretreatment triggered chromosomal aberrations, aneuploidy, binucleate cells, chromosome deletion, deformed nuclei, micronuclei, chromosome fragmentation, and sticky chromosomes during the metaphase and anaphase stages of the cell cycle. Furthermore, when compared to control, in NPs subjected plants' the mitotic index was significantly increased. The increased frequency of mitotic abnormalities caused by AgNPs, including chromosome divergence, lagging chromosome, nuclear disintegration, chromosome fragmentation, pole distortion in anaphase and C-metaphase, is mainly due to its effects on mitotic spindles and chromosome orientation changes at different stages of the cell cycle. The function of the mitotic spindle is impaired as a result of AgNPs interaction with a highly reactive tubulin-SH group [236].

When compared to control plants, silver nano-priming enhanced seed germination, shoot length, fresh and dry biomass, and decreased the respective root parameters in wheat and barley. Chromosome aneuploidy, binucleate cells, deletion chromosomes, deformed nuclei, micronuclei, chromosome fragmentation, and stickiness chromosomes are all caused by AgNPs priming. Thus, AgNPs may be able to pass through inside plants, causing damage to cell division stages and causing chromosomal disruptions [237]. The entry of nano-Ag into the cell might have induced DNA harm [238], or it could have been caused by the inhibition of DNA synthesis during the S-phase [239]. In the three plants exposed to AgNPs, structural chromosomal aberrations and nuclei deformations were detected. Ag and ZnO NPs disrupted cell division stages, interrupted metaphase, caused multiple chromosomal breaks, and caused cell disintegration in onion root tips [128, 240]. Nanoparticles have the ability to interrelate with and affect the function of protein kinases and their families in signal transduction, and they are also involved in the regulation of cell cycle events such as DNA replication and cell division [241]. TiO_2_ nanoparticles interrupted one of the mitotic checkpoints- PLK1 protein and its function. These proteins regulate and control the mitosis process, which includes contractile ring formation and cytokinesis.

### Priming-induced molecular responses against abiotic stresses

Nano-priming is a technique that uses NPs to boost seed germination and growth, and it has been shown to produce significant results [58]. Seed priming is a technique of promoting seed germination and enhancing seed tolerance to abiotic and biotic stresses. According to Chen and Arora, the abiotic stress caused by seed priming during germination could activate stress-reacting systems in seeds, improving tolerance through germination and seedling development [242]. Drought is a major ecological concern that affects crop productive output and nutrient content, ultimately affecting human nourishment. The investigations have shown that MWCNT and AgNPs can assist a plant to withstand drought and saline stress [215]. The seed priming of *Alnus subcordata* (*Caucasian alder*) with MWCNTs showed drought resistance and exhibited increased germination percentage, SVI, enhanced the length of root and shoots [93]. Dimkpa et al. reported the influence of drought on the acquisition and transfer of nutrients in sorghum but whether ZnO NPs could mitigate such effects [243]. Drought delayed the emergence of flag leaves and grain heads for 6–17 days, but the delays for ZnO NPs have been reduced to five days. In addition, drought lowered (76%) grain yield, but the ZnO NPs application enhanced the yield (22–83%) upon drought. ZnO NPs promote plant growth, increase the yield, strengthen important food grains with Zn nutrients, and improve N acquisition during drought stress. Zn-based fertilizers have been shown to play a critical role in relieving wheat plant drought stress through Zn-mediated increases in photosynthesis pigment, reactive oxygen scavenging substances, and lipid peroxidation reduction [244]. Various methodologies for Zn fertilization are used, which include foliar spraying and soil mixing [244]. This has significant potential for developing crop production system resilience, enduring human/animal food safety, minimizing nutrient loss, and reducing environmental pollution caused by N-fertilizers. The drought-induced negative effects were counteracted by the Cu, Zn-NPs in wheat seedlings. The enhanced antioxidant enzyme activity reduced the MDA level and sustained the photosynthetic pigments and enhanced the relative water content potential in NP-treated plants [82, 221]. In maize, priming with Cu NPs showed a favorable response to drought stress through improved leaf water content and plant biomass. In addition, enhanced anthocyanin, chlorophyll, carotenoids, total seed number and grain yield were observed in Cu NPs primed plants in drought stress treatment [83]. They conclude that Cu NP-mediated protection against drought stress is a promising tool for the generation of improved water deficit tolerant crops.

The supplementation of TiO_2_ NPs alone or the synergistic application with sodium nitroprusside potentially ameliorates the PEG-induced drought stress in wheat seedlings. Priming considerably enhanced seed germination percentage, root and shoot length, SVI, shoot and root fresh biomass compared to control, drought-stressed plants without priming [87]. The seeds of marigold (*Calendula officinalis* L.) primed with silicon NPs under drought stress exhibit enhanced antioxidant activity, quercetin, and total flavonoid content compared to control plants. Therefore, the priming with nano silicon under drought stress might enhance the physiological and metabolic traits of *Calendula officinalis* L. [245]. Silica or silicon dioxide NPs (SNPs) utilized to rectify heavy metal phytotoxicity have been studied previously [246]. Aluminum (Al) toxicity has become the main hindrance to plant growth in acid soils. The impact of Al solely or combined with SNPs on Al accumulation and detoxification, plant growth, photosynthetic C assimilation and redox homeostasis has been examined [247]. Al accumulation in maize organs reduced their growth, impacted photosynthesis and elevated ROS production, through induced NADPH oxidase and photorespiration activities, and cell damage was more evident in roots than in leaves. Co-application of SNPs considerably reduced the action of photorespiratory enzymes and NADPH oxidase. Antioxidant defense systems were activated at enzymatic and nonenzymatic levels. Furthermore, increased organic acid accumulation and metal detoxification in roots were generated by SNPs as a shielding mechanism against Al toxicity [247]. SNPs enhanced ascorbate (ASC) and glutathione (GSH) content, providing a strong defense for plant organs. Si and SNPs could reduce arsenate toxicity in maize plants by enhancing the components of the ASC-GSH cycle [248]. SNP-induced GSH levels have previously been proposed as a mechanism to protect plants from oxidative stress [223]. Silica NPs appear to be a natural choice for the development of pest-control agriproducts because silicon has already been used to improve plant tolerance to a variety of abiotic and biotic stresses [249]. Application of ZnO NPs potentially ameliorated the Cd-induced toxicity and increased plant growth, photosynthetic rates, and stomatal conductance in tomato. It also enhanced the protein content, nitrate reductase and carbonic anhydrase activities in ZnO NPs treated plants [87].

Biosynthesized AgNPs (as a priming agent; 1 mg/L) reduce salt-induced toxic effects in germinating wheat grains grown in salt stress (25 and 100 mM NaCl). In general, AgNPs priming promotes wheat grain germination and growth. Furthermore, it influences plant hormone balance by increasing IBA, NAA, BAP, and decreasing ABA content [250]. Salt stress inhibited seedling growth in *Triticum aestivum*, as evidenced by a marked decline in growth performance index, pigment contents and stability index, auxins and cytokinin levels, and a significant enhancement in ABA, particularly at 100 mM NaCl. AgNPs priming significantly improved all of these parameters, particularly growth parameters and photosynthetic efficiency, as well as phytohormone balance, implying that AgNPs priming may play a role in improving plant tolerance to environmental stresses such as salinity [250]. Manganese oxide nanoparticles (Mn NPs) or MnSO_4_ primed seeds of *Capsicum annuum* in water (0 mM sodium chloride) resulted in a root that was 33 percent longer on average than the absolute control [122]. In the control group, root elongation was inhibited by 57% when salt stress (100 mM sodium chloride) was practiced. As a result, priming seeds with manganese sulfate or Mn NPs improved root growth under water germination conditions and partly alleviated the antagonistic effects detected by salt stress spectroscopy [122]. According to Cuajungco et al. [251], protein N–H bond strength may have been changed owing to chelation with metal, in this case, MnNPs or MnNPs dissolved ions (Mn^2+^), which could be the metal "donors."Ye et al. also published those priming seeds with MnNPs prior to salt stress exposure (100 mM NaCl) altered the N–H bond vibration in *C. annuum* L. [122]. Moreover, uptake of Mn was inhibited by salt stress, thus inducing Mn deficiency in plants, thereby affecting cell division and plant growth [252]. Also, when Mn was supplemented externally during a priming process, it effectively accumulated Mn inside the seeds. MnSOD expression was altered by priming conditions (P ≤ 0.001), salt stress (P ≤ 0.001), and changes in mRNA were influenced by the Mn concentration and type used during the priming process. The positively charged MnNPs spectra might well be connected to SOD up-regulation [122]. Silica NPs of two different types (SiNPs 50 & 100 nm) were used for priming in wheat. This led to enhanced seed germination, which is higher in SiNPs of 50 nm in size. Moreover, the SiNPs primed, and control seedlings were subjected to salt stress (100 mM NaCl) and the growth traits such as biomass and chlorophyll contents were found to be higher in the SiNPs priming. This indicates that seed germination and salinity tolerance in wheat can be improved via priming with SiNPs [253]. Yeo et al. noted that treatment with Si NPs reduced sodium uptake and transpiration bypass flow in *Oryza sativa* [254]. Latef et al. found that priming Lupin seeds with ZnO NPs increased the growth of salt-stressed (150 mM NaCl) plants by increasing the number of photosynthetic contents, total phenols, organic solutes, ascorbic acid, and zinc [91]. Moreover, nano-priming enhanced the SOD, CAT, POD, and APX enzyme activities as well as reduced the malondialdehyde (MDA) and sodium (Na) levels as compared to salt-stressed plants without nano-priming. Thereby, priming the seeds with ZnO NPs enhanced the salinity tolerance in *Lupinus termis* plants. Similarly, Sharma et al. used 20–40 mg/L green ZnO NPs to prime-aged seeds of a pre-flowering homozygous mutant (BM6) of Pusa basmati (*O. sativa*) that vastly enhanced germination and seedling vigour, particularly in comparison to zinc sulphate (ZnSO_4_) priming and conventional hydropriming [255]. The sorghum seeds soaked with a lower level of Fe_2_O_3_ NPs (10 mg/L) potentially induce a rapid and higher germination rate, whereas the seedling growth was higher at 50 and 100 mg/L of Fe_2_O_3_ nanoparticles. The higher concentration of Fe_2_O_3_ NPs (500 mg/L) seed priming enhanced the plant growth via improved photosystem II efficiency, chlorophyll index, photosynthesis, and relative H_2_O content as well as the reduced MDA level. Their results suggest that seed priming with Fe_2_O_3_ NPs could increase the germination rate and development of young plants as well as give protection against salinity-induced damage [256]. The poly (acrylic acid) coated cerium oxide (CeO)NPs primed and control cotton seeds were germinated in salt stress (200 mM NaCl) and the CeO NPs primed seeds exhibits enhanced seedling root length, fresh and dry mass, improved root vitality under salt stress than control (water) seedlings. The roots of CeO NPs primed seeds revealed similar Na levels, considerably reduced potassium (6%), enhanced calcium (22%) and magnesium (60%) compared to control. As compared to control (no NP treatment), the poly (acrylic acid) coated CeO NPs exhibit differential expression of 4779 root transcripts in salt stress, which primarily includes the genes corresponding to ROS pathways and ion homeostasis, suggesting that reactive oxygen species and conserved Ca^2+^ transduction cascades may possibly play crucial roles in salt stress resistance imposed by poly (acrylic acid) coated CeO NPs [209].

There are intensive discussions on possible mechanisms concerning the effect of metal nanoparticles on photosynthetic apparatus (PSA). For instance, the ability to improve the absorption of light by chlorophyll molecules via the plasma resonance effect is associated with the promotion by NPs of light-captured photosynthetic phase photochemical reactions [257]. Metal NPs can eliminate PSII chlorophyll from excessive excitation, absorb the energy of excited electrons [187] and act as a kind of "protector" against oxidative stress. The literature shows that NPs can reduce the production of ROS in plants. For example, in spinach, titanium NPs are able to protect chloroplasts from intensive aging owing to oxidative stress, while the ROS level in the leaves decreases at the same time [258]. Metal NPs not only affect antioxidant defense system (AOS) enzymes but also encourage the accumulation of antioxidant-proline, glutathione and carotenoids in plant tissues [259]. It has also been reported that nanostructured SiO_2_ significantly reduces plant transpiration rate and improves plant green coloration and shoot expansion [260]. Fe_3_O_4_ NPs (1–4 mg/L) stimulate less genotoxicity and induce growth and development in rocket seedlings. At lower concentrations, it slightly enhanced the chlorophyll fluorescence and significantly enhanced the *miR159c* expression [261]. In tobacco, TiO_2_ was shown to induce *miR159* slightly [262] and *miR159* is crucial for plant growth and environmental stress responses.

#### Impact against biotic stresses

Nanoemulsions are the combination of water and oil phase systems like pesticide formulations to control the pest population in agronomy [263]. PEG NPs confined with garlic energetic oil are used to control red flour beetle insects that are widely observed in stored foods. The spongy hollow silica NPs called porous hollow silica nanoparticles (PHS NPs) loaded with validamycin pesticide is an accomplished in-controlled delivery system that benefits agriculture [264]. Most charged nano-silica (3–5 nm) can be effectively used in agriculture to control ectoparasites of animals and insects. Silicon has already been of great interest in combating several biotic and abiotic stresses, and hence, silica NPs will be a wonderful alternative to produce agri-related products against pests [249]. Metal NPs including Ag, Cu, ZnO, and TiO_2_ have been continuously scrutinized for their antibacterial, antifungal and antiviral activities [61, 66, 265, 266]. Alumina nano-insecticides were effective against rice weevil and a lesser grain borer; these pests are transported from one area to another in foodstuffs [267]. By using a well diffusion assay, nanostructured silver was found to have the best antifungal activity against *Alternaria alternata*, *Sclerotinia sclerotiorum*, *Macrophomina phaseolina*, *Rhizoctonia solani*, *Botrytis cinerea*, and *Curvularia lunata* [268]. The sun-hemp rosette virus was completely suppressed when AgNPs were sprayed onto bean leaves, as reported [269]. Elbeshehy et al. also demonstrated that silver NPs sprayed 24 h after infection on *Vicia faba* infected with bean yellow mosaic virus (YMV) produced significant results before infection synchronous at the time of inoculation [270]. Foliar spray of AgNPs (5 g/mL) may have decreased the number of fungal spores, implying that biosynthesized silver NPs are effective against fungal spore formation. The number of lesions decreased from 2.9/leaf in pathogen-infected plants to 0.9/leaf in silver nanoparticle treated plants, indicating that green synthesis of silver nanoparticles might enhance plant productivity and reverse the 10–30% damage. Similarly, use of phytogenic AgNPs, results in advanced seed germination, plant development, chlorophyll, carotenoids, and protein content, as well as antifungal activity against *Aspergillus niger* in important agricultural crops like *Oryza sativa*, *Zea mays*, and *Arachis hypogaea* [270]. The green synthesized AgNPs using fungal extracts of *Trichoderma harzianum* and *Aspergillus fumigatus* were used to treat the seeds of tomato that showed improved growth traits (plant height, fresh & dry biomass, yield). In addition, AgNPs potentially suppressed the occurrence of bacterial canker disease [271]. Chitosan NPs have antimicrobial properties, such as the ability to control *Fusarium* crown, root rot in *Solanum lycopersicum*, Botrytis bunch rot in *Vitis vinifera*, and *Phyricularia grisea* in *O. sativa*, [272] but they are less effective against bacteria. Malerba and Cerana [273] studied chitosan's antimicrobial effects, agglutination reactions, cell membrane disruption, suppression of H^+^-ATPase activity, inactivation of toxin production and microbial growth, suppression of mRNA and protein synthesis, and nutrient flow. Improved SVI and enhanced defense responses were seen in the tomato and maize seeds primed with Cu-chitosan NPs [274]. In addition, Cu-chitosan NPs treated maize seeds stimulate the amylase and protease enzymes [275]. The seeds primed with nano chitosan exhibit activity against seed-borne pathogens [275]. The improved restricted blast disease occurrence was noticed in finger millet plants treated synergistically with Cu-chitosan NPs, compared to foliar spray alone [276]. Furthermore, the synergistic and foliar spray of Cu-chitosan NPs in finger millet stimulates various defense-related enzymes like chitinase, chitosanase, β-1,3 glucanase, peroxidase, polyphenol oxidase, and protease and exhibits improved resistance to the blast fungus *Pyricularia grisea* [276]. Henceforth, the pretreatment of nano-pesticides could promote growth and reduce the incidence of disease occurrence in plants.

### Distribution of minerals

Metal and metal oxide NPs are stated to alter the mineral nutrition profile of many important crops. Ag NPs on foliar feeding decreased mineral elements in different parts of *S. lycopersicum* seedlings that exhibited nutrient deficiency symptoms [277]. In another article, it was quoted that, the potassium was increased whereas magnesium, phosphorus, and sulphur were decreased on metal NPs application [278]. In transgenic *Gossypium hirsutum*, cerium dioxide and silicon dioxide nanoparticles completely altered the mineral content in shoots and roots [279, 280]. Similarly, there was no modification in the mineral profile of CeO_2_ NPs in many parts of radish, but decreased Ni was observed with enhancing nanoparticles in the soil. The level of Ca, Fe, and Zn were decreased after CuO NPs exposure in beans, while sodium (Na) concentration was increased. Furthermore, in lettuce, nanostructured CuO reduced the contents of Ca, Mn, P, and Mg. CuO nanoparticles (5, 10, and 20 mg/L) improved copper, phosphorus, and sulfur concentrations in alfalfa shoots by 100%, 50%, and 20%, respectively, while P and iron concentrations were reduced in lettuce shoots [104]. The concentrations of K, Ca, Mg and S were reduced after SnO_2_ NPs exposure in the leaves and stems of tomato [278]. The soluble metals released from NPs might be a possible reason for the modified or reduced mineral uptake in many plants [52]. NPs can be used to enhance mineral nutrients, particularly micronutrient (Zn, Fe, Mn, B etc.) concentrations, in plants, which may positively influence plant growth under certain conditions [281, 282]. For example, foliar spray of SiO_2_ NPs increased the translocation of potassium (K), magnesium (Mg), and Fe from the uppermost nodes to rachises of rice, whereas SiO_2_ NPs had no significant effects on the translocation of calcium (Ca), zinc (Zn), and manganese (Mn) [283]. Antioxidant defense system and leaf senescence were also delayed by MWCNTs treated plants. In an appealing way, the nutritional profile analyzed through inductively coupled plasma-optical emission spectrometry in the leaves and kernels of MWCNTs-treated plants were found to be higher than control plants, indicating that MWCNTs could alter nutrient distributions [47].

Generally, in plants, mineral uptake and accumulation are reduced during metal and metal oxide nanoparticles exposure. Adsorption, absorption, and transport from the root surface to the xylem, as well as translocation from the roots to the shoots or grains, are the mechanisms by which NPs reduce mineral uptake. Salinity is one of the factors that inhibit Mn uptake and initiate Mn deficiency in plants, which was controlled in this study by additional Mn supplementation from Mn nanoparticles and MnSO_4_ [122]. Moreover, Mn exists as a major component in the protein complex, essential for performing water oxidation during photosynthesis. Although this is a one-way transportation process, Na is stored in a huge quantity in shoots rather than roots. Therefore, during salt stress, MnNPs primed seeds (0.1 and 1 mg/L) exhibited a positive Na redistribution, whereas Mn^2+^ primed seeds did not show any effective changes. During salt stress, a steady increase in the calcium content was observed in shoot and root at all priming conditions (hydro, MnSO_4_, MnO NP). Since calcium is necessary for leaf tissue development, its accumulation was usually observed in the shoot [122]. Ca, as previously discussed, is not only important for plant cell wall and membrane function, but it also functions as a secondary messenger in signal transduction in plant cells. Therefore, Ca^2+^ is extremely essential for cellular communication, especially when the shoot is completely accumulated with Ca^2+^ ions.

Drought reduced grain nitrogen translocation by 57% and total nitrogen acquisition in the root, shoot, and 22% of grain. Furthermore, according to reports, ZnO nanoparticles (5 mg/kg) enriched (84%) grain nitrogen translocation compared to the drought control and restored total nitrogen levels to pre-drought levels [243]. Drought conditions increased phosphorus uptake in the shoot (39%) while restricting grain phosphorus translocation (63%). ZnO-NPs adjustment (5 mg/kg) to drought-affected plants developed total potassium attainment (16–30%) and grain potassium (123%), relative to the drought control. Drought lowered (32%) average grain zinc concentration; though, zinc oxide NPs amendments improved (94%) grain zinc under drought [243]. Similarly, the iron was improved considerably in the shoots and roots of primed young plants after nZVI priming. Results suggested that a low concentration of nZVI (< 40 mg/L) acted as an effective priming agent for the seeds with improved plant growth at its later stage [28]. These results suggest that the minerals distributed showed a consequent impact on NP treatments and that it varied according to the type of NPs and treatment.

## Conclusion and future perspective

The increasing consumption of nanomaterials demands larger production. Several nanomaterials, including metal and metal oxide NPs, were used in nano-priming to improve seed germination and develop resistance to various stresses. Like other priming techniques, nano-priming induces synchronized germination, stimulates plant growth and augments tolerance to abiotic stresses. Numerous reports have shown that the induction of ROS by NPs is the basis to produce secondary metabolites. ROS acts as signaling cues in plant defense mechanisms against biotic and abiotic stresses that lead to the induction of stress-specific secondary metabolites. However, comprehensive knowledge on NP adhesion and phytohormone crosstalk in nano-priming induced seed germination and the signaling cascades that participate in secondary metabolite production during nano-priming are still far behind. Thus, it mandates a complete genome-wide transcriptome study in different nano-priming conditions will be useful in understanding the commonly controlled networks responding to NPs. Moreover, during nano-priming treatments, the utilization of various aquaporin family gene mutants is probably useful to dissect additional transcription co-factors correlated with the expression of aquaporin genes in primed seeds. Furthermore, it is noteworthy to identify the intercellular trafficking of phosphorylation-dependent PIP and TIP aquaporin induced by NPs in nano-priming that leads to enhanced water uptake.

## Data Availability

Not applicable.

## Abbreviations

NPs: Nanoparticles
ROS: Reactive oxygen species
AuNPs: Gold nanoparticles
ABA: Abscisic acid
GA: Gibberellic acid
SVI: Seed vigour index
PEG: Polyethylene glycol
IAA: Indole acetic acid
CNPs: Carbon nanoparticles
MWCNTs: Multi-walled carbon nanotubes
CNTs: Carbon nanotubes
TFs: Transcription factors
SOD: Superoxide dismutase
CAT: Catalase
K: Potassium
Mg: Magnesium
P: Phosphorus
S: Sulphur
SWCNHs: Single-walled carbon nanohorns
TAA: Total antioxidant activities
DPPH: 2,2-Diphenyl-1-picrylhydrazyl
PGPRMs: Plant growth-promoting rhizomicrobes
POD: Peroxidase (POD)
APX: Ascorbate peroxidase
nZVI: Zero-valent iron nanoparticles
PA: Phosphatidic acid
LPC: Lysophosphatidylcholine
CL: Cardiolipin
LPE: Lysophosphatidylethanolamine
PE: Phosphatidylethanolamine
PC: Phosphatidylcholine
PI: Phosphatidylinositol
SQDG: Sulfoquinovosyl diacylglycerols
PG: Phosphatidylglycerol
DGDG: Digalactosyldiacylglycerol: PS: Phosphatidylserine
MGDG: Monogalactosyl diglyceride
JA: Jasmonic acid
SA: Salicylic acid
ET: Ethylene
BRs: Brassinosteroids
MAPK: Mitogen-activated protein kinase
CaBPs: Calcium-binding proteins
PAL: Phenylalanine ammonia-lyase
CHS: Chalcone synthase
TPC: Total phenolic content
TFC: Total flavonoid content
RAS: Rosmarinic acid synthase
HPPR: Hydroxyphenyl pyruvate reductase
HRCs: Hairy root cultures
MI: Mitotic index
CA: Chromosomal aberrations
PSA: Photosynthetic apparatus
PHSNPs: Porous hollow silica nanoparticles
